# M2 macrophage-secreted exosomes promote metastasis and increase vascular permeability in hepatocellular carcinoma

**DOI:** 10.1186/s12964-022-00872-w

**Published:** 2023-10-30

**Authors:** Yiwei Lu, Guoyong Han, Yao Zhang, Long Zhang, Zhi Li, Qingyuan Wang, Zhiqiang Chen, Xuehao Wang, Jindao Wu

**Affiliations:** 1 Department of Hepatobiliary Surgery, The First Affiliated Hospital of Nanjing Medical University, 300 Guangzhou Road, Nanjing, 210029 China; 2 Key Laboratory of Liver Transplantation, Chinese Academy of Medical Sciences, NHC Key Laboratory of Living Donor Liver Transplantation,, Nanjing Medical University Nanjing, Nanjing, Jiangsu Province China; 3 Department of General Surgery, The First Affiliated Hospital of Nanjing Medical University, Nanjing, China; 4 State Key Laboratory of Reproductive Medicine, Nanjing Medical University, Nanjing, China

**Keywords:** M2 macrophages, Exosomes, EMT, Angiogenesis, Vascular permeability, miR-23a-3p

## Abstract

**Background:**

Metastasis is a key feature of malignant tumors and significantly contributes to their high mortality, particularly in hepatocellular carcinoma (HCC). Therefore, it is imperative to explore the mechanism of tumor metastasis. Recently, tumor-associated macrophages (TAMs) have been demonstrated to promote tumor progression, while TAM-derived molecules involved in HCC metastasis warrant further investigation.

**Methods:**

THP-1 was treated with IL-4 (Interleukin-4) and IL-13 (Interleukin-13) for M2 polarized macrophages. Exosomes derived from M2 macrophages were characterized. Then, HCC cells or human umbilical vein endothelial cells (HUVECs) were co-cultured with M2 macrophages or treated with M2 macrophage-secreted exosomes. Next, Transwell®, Scratch assay, tube formation, and endothelial permeability assays were performed. Moreover, RT-PCR, western blotting, immunofluorescence, and ELISA were used to assess mRNA and protein expression levels. Finally, the miRNA expression profiles of exosomes derived from M2 and M0 macrophages were analyzed.

**Results:**

M2 macrophage infiltration was correlated with metastasis and a poor prognosis in HCC patients. M2-derived exosomes were absorbed by HCC and HUVEC cells and promoted the epithelial-mesenchymal transition (EMT), vascular permeability, and angiogenesis. Notably, MiR-23a-3p levels were significantly higher in M2-derived exosomes and hnRNPA1 mediated miR-23a-3p packaging into exosomes. Phosphatase and tensin homolog (PTEN) and tight junction protein 1 (TJP1) were the targets of miR-23a-3p, as confirmed by luciferase reporter assays. Lastly, HCC cells co-cultured with M2-derived exosomes secreted more GM-CSF, VEGF, G-CSF, MCP-1, and IL-4, which in turn further recruited M2 macrophages.

**Conclusions:**

Our findings suggest that M2 macrophage-derived miR-23a-3p enhances HCC metastasis by promoting EMT and angiogenesis, as well as increasing vascular permeability.

Video Abstract

**Supplementary Information:**

The online version contains supplementary material available at 10.1186/s12964-022-00872-w.

## Background

Hepatocellular carcinoma (HCC) is one of the most common malignant tumors, with about 800,000 new cases diagnosed yearly. In addition, it has a high mortality rate of approximately 700,000 deaths each year [1, 2]. Regarding early HCC, surgical resection or liver transplantation can achieve a favorable prognosis, but some patients lose surgical opportunities when intrahepatic or distant metastases develop. As is well documented, metastasis is one of the predominant causes of cancer-related deaths in HCC [3, 4]. Therefore, exploring the mechanism of metastasis is vital for improving the survival of individuals with advanced HCC.

The tumor microenvironment comprises tumor cells, immune cells, interstitial cells, and secreted active mediators [5, 6]. In the tumor microenvironment, interactions among various types of cells reshape each other and maintain the emergence and development of tumors. Among the numerous mutant cells infiltrated in the tumor environment, tumor-associated macrophages (TAM) constitute the majority, accounting for 30%–65% of cells in HCC [7–9]. Macrophages can be activated in several microenvironments with distinct properties and exhibit a wide range of activated phenotypes and functions. M2 macrophages highly express arginase 1 (Arg1), macrophage mannose receptor (MMR), and scavenger receptors [10, 11]. According to past studies, TAMs promote tumor progression through immunosuppression. In HCC, TAMs secrete more transforming growth factor-beta 1 (TGF-beta 1) and induce HCC cells to develop stemness [12].

Exosomes are 50–100-nm lipid bilayer microvesicles secreted by the body cells under physiological and pathological conditions. They can encapsulate proteins, nucleic acids, and lipids to transport substances between cells, thereby affecting the function of recipient cells [13]. Studies have reported that exosomes play a crucial role in tumors. In a hypoxic environment, tumor cells secrete exosomes with miR-301a and activate the PTEN/PI3K signaling pathway to induce M2 polarization of macrophages, thereby promoting pancreatic cancer metastasis [14]. The acidic microenvironment in HCC induces higher expression levels of miR-21 and miR-10b in tumor cells and triggers cancer cell proliferation and metastasis [15]. However, whether M2 macrophage-derived exosomes promote HCC metastasis requires further investigation.

This study aimed to investigate the effect of M2 macrophage-derived exosomes on the metastasis of HCC cells. The results illustrated that M2 macrophage infiltration was significantly higher in HCC tissues with metastasis compared with non-metastatic ones and was negatively correlated with prognosis. M2 macrophage-derived exosomes promoted HCC metastasis by transmitting miR-23a-3p into HCC cells and HUVECs to induce EMT and angiogenesis, as well as increased vascular permeability. Our study provides a new theoretical basis for the treatment of HCC metastasis.

## Materials and methods

### Cell lines and culture

The HCC cell lines Huh-7 and SMMC-7721 were utilized in this study. The cell lines were provided and identified by our laboratory for living donor liver transplantation. The cells were cultured in Dulbecco’s modified Eagle medium (DMEM; Gibco, Grand Island, NY, USA) containing 10% fetal bovine serum (Gibco), 100 U/mL penicillin, and 100 μg/mL streptomycin (Invitrogen, Carlsbad, CA, USA) at 37 °C in a 5% CO_2_ environment. RPMI-1640 (DMEM; Gibco) containing 10% fetal bovine serum, 100 U/mL penicillin, and 100 μg/mL streptomycin was used for THP-1 cells. For M2-polarized macrophages, THP-1 cells were treated with 5 ng/mL PMA (Sigma, USA) for 24 h and then incubated with 20 ng/mL IL-4 plus 20 ng/mL IL-13 (PeproTech, USA) for 48 h. They were then identified by microscopy, flow cytometry, and immunofluorescence.

### Isolation of macrophage exosomes

M2 macrophages acquired from THP-1 were incubated with 10% exosome-free FBS for 48 h. Then, the medium was collected and centrifuged at 1,000 g for 5 min at room temperature and subsequently at 15,000 g for 15 min at 4 °C, followed by filtration with a 0.22-μm membrane to remove cells, cell fragments, and organelles. Next, the supernatant was centrifuged at 110,000 g for 70 min at 4 °C. The precipitate was then collected and resuspended in PBS. The exosomes were characterized by transmission electron microscopy and western blotting.

### Transmission electron microscopy (TEM)

For TEM, the exosomes were resuspended in PBS, dropped onto a copper mesh, and stained with 1% uranyl acetate. The mesh was dried for 5 min and observed under a transmission electron microscope (FEI, Tecnai G2 spirit).

### Exosome tracking

The extracted exosomes were incubated with 0.5 mL of diluent C. Approximately 4 L of a PKH67 dye solution were incubated with 0.5 mL of diluent C. The exosomes and PKH67 were mixed and incubated overnight at 4 °C. Afterward, the mixture was rinsed with PBS to remove excess dyes. The prepared dye solution was seeded to the prepared cells for 24 h of incubation. Finally, images of the cells were captured under a laser confocal microscope.

### Cell transfection

For the lentivirus (Genepharma, Shanghai, China), cells were plated into six-well plates at a density of 2 × 10^5^ cells per well. When the density reached 30%–40% after incubation, the lentiviruses were transfected into the HCC cells. When cell confluency reached 80%–90%, the cells were transferred into 100-mm dishes and selected for 2 weeks using puromycin (10 μg/mL). For shRNA, Lipofectamine 3000 (Invitrogen) was used according to the manufacturer’s protocol. Briefly, the cells were plated in six-well plates at a density of 1.5 × 10^5^ cells per well one day before transfection. When the cells were in the log phase, a mimic was added to each well. The medium was replaced after incubation for 6 h. The cells were harvested after 48–72 h for subsequent experiments. All sequences of the siRNA were listed in Additional file 1: Table S4.

### RNA isolation and quantitative real-time PCR

Total RNA was extracted from human specimens and cells using TRIzol reagent (Takara, Dalian, China) according to the manufacturer’s instructions. RNA quality and concentration were evaluated with a Nanodrop 2000 system (NanoDrop Technologies, IL, USA). The cDNAs of miRNA were reverse transcribed using the reverse translate kit PrimeScript RT Master Mix (DRR037A; Takara) according to the manufacturer’s protocol. Sequence-specific primers for U6 and miR-23a-3p were synthesized by RiboBio (Guangzhou, China). Real-time polymerase chain reaction was performed with SYBR Premix ExTaq (TaKaRa, Dalian, China) and the ABI Prism 7900HT (Applied Biosystems, Foster City, CA, USA). Glyceraldehyde-3-phosphate dehydrogenase (GAPDH) was used as the internal control for mRNA quantification. The relative expression was calculated using the 2^–ΔΔCT^ method. The primer sequences used in real-time PCR are displayed in Additional file 1: Table S2.

### Western blotting

Cells and tissue specimens were lysed using a lysis buffer (10 mM Tris, 1 mM EDTA, 1 mM DTT, 60 mM KCl, 0.5% v/v NP-40, and protease inhibitors) on ice. Protein samples (20 μg) were separated using 10% sodium dodecyl sulfate–polyacrylamide gel electrophoresis (SDS-PAGE) and transferred onto a polyvinylidene difluoride membrane. After blocking, the bands were incubated with specific antibodies. Proteins were visualized with an enhanced chemiluminescence detection kit according to the manufacturer’s recommendations (Beyotime, Shanghai, China). Relative protein levels were calculated based on GAPDH levels. The antibodies used in the study are presented in Additional file 1: Table S3.

### Immunohistochemistry

A diaminobenzidine detection kit (Maixin-Bio, Fuzhou, China) was employed for immunohistochemical staining according to the manufacturer’s instructions. The dissected HCC and peritumor tissues were fixed and embedded in paraffin. Then, 5-μm thick consecutive sections were cut and mounted on glass slides, dewaxed, rehydrated, and the antigen was retrieved. The sections were incubated with a primary antibody at 37 °C for 1 h, a biotin-labeled secondary antibody for 10 min, and a streptavidin–peroxidase conjugate for 10 min. A 0.02% diaminobenzidine solution was used as a chromogen to visualize peroxidase activity. The sections were lightly counterstained with hematoxylin, mounted with Permount, and examined under light microscopy.

### *Transwell*

Migration and invasion analysis was performed using Transwell chambers (Corning Incorporated, Corning, NY, USA). For migration, following suspension in a serum-free medium, 3 × 10^4^ cells were seeded into the Transwell insert supplemented with DMEM containing 5% serum. After 48 h of incubation, the cells on the bottom side of the membrane were fixed with 95% alcohol and stained with crystal violet for 20 min at room temperature. Next, the number of cells on the lower side of the filter was counted under a microscope. Each experiment was performed in triplicate. For HUVEC migration, 1 × 10^4^ cells were seeded and then incubated for 24 h. For invasion, Matrigel was plated on the upper surface before cell inoculation. The other steps were identical to those in the migration assay.

### Scratch assay

HCC cells were seeded in 6-well plates, and when covered with a layer of cells on the 6-well plate, a cross was scratched at the center using a 200 L plastic pipette tip and cultured in a serum-free medium for 0 h and 48 h. Afterward, the floating cells in the upper layer were washed with PBS, photographed under an inverted microscope, and the percentage migration area was calculated.

### *Endothelial permeability assay *in vitro

Endothelial permeability assay was performed according to a previous study [16]. Briefly, 2 × 10^4^ HUVEC cells were seeded into 24-well Transwell filters (8-μm pore size; Corning) and then incubated when cell confluency reached 100%. Afterward, the 24-well Transwell filters were carefully washed with PBS. Finally, 20 mg/mL rhodamine-dextran (average MW: ~ 70,000; Sigma-Aldrich) was added into the upper chamber, and after 1 h of incubation, the lower medium was collected and assessed under laser confocal scanning microscopy.

### Transendothelial invasion assay

A transendothelial invasion assay was conducted according to a previous study [17]. Endothelial monolayers were prepared as described earlier. The 24-well Transwell filters were placed into new 24-well plates, and 5 × 10^4^ of GFP-positive HCC cells were subsequently seeded into the upper chamber, while the lower chamber was filled with 750 μL of 10% exosome-depleted FBS-SFM. After 12-h incubation, the cells in the upper and lower chambers were collected and counted under a fluorescent microscope.

### Tube formation assay

Growth factor-reduced Matrigel (BD Biosciences, San Jose, CA, USA) was diluted with a serum-free medium in a 1:1 ratio, and then 300 L was placed into each well of the 24-well plates and incubated for 30 min. The different groups of HUVEC cells were suspended at a density of 1.2 × 10^6^, and 100 μL of the cell suspensions was seeded into the 24-well plates. After 4–6 h of incubation, tube images were captured using a digital camera, and tube formation was determined using the ImageJ software.

### Chick chorioallantoic membranes (CAM)

All eggs were cultured at 37℃ for about 7 days, we then open a window on the shell of the fertilized eggs to expose CAM, and then the surface was covered with filter paper plates with different treatment groups. Next, cover the window with duct tape for further incubation. 2–3 days later, the CAM was fixed in formaldehyde to take pictures.

### In vivo* metastasis model*

Lung metastasis model was established to examine the effect of M2 exo-miR-23a-3p on tumor metastasis in Vivo. 5-week-old male mice were then injected with 5 × 10^6^ HCC cells containing 150 μl PBS. After 6–7 weeks, the mice were killed, tumor nudes were counted, photographed, collected and embedded in paraffin.

### Immunofluorescence assay

For immunofluorescence, cells were fixed with 4% formaldehyde, permeabilized with 0.2% Triton X-100, and blocked with 1% bovine serum albumin in PBS. The cells were incubated with the primary antibody overnight at 4 °C and then incubated with FITC-conjugated goat anti-rabbit IgG after three washes. Finally, the cells were washed and mounted with a mounting medium containing 4,6-diamidino-2-phenylindole (DAPI). Images were captured with a scanning microscope.

### ELISA

Briefly, the cell culture supernatant was collected, centrifuged at 1,000 g at 4 °C for 30 min, and the supernatant was stored at -80 °C until use. The samples to be tested were thawed on ice. Standard wells were set up, and then 50 L of pre-replaced standards at different concentrations were added to each well. Next, 50 L of each sample to be tested was added to each well. Thereupon, 50 L of horseradish was added per well and incubated for 1 h at 37 °C. The liquid was later discarded, and 300 L of cleaning solution was added. After 2 min, the cleaning solution was discarded, and the microwell plate was patted dry on the absorbent paper to remove any residual liquid. The microwell plate was rinsed another 5 times, and then 50 L of the substrate was added to each well and incubated for 15 min. Lastly, 50 L of a stop solution was added to terminate the reaction, and the absorbance of each well was assessed at a wavelength of 450 nm.

### CCK-8 assay

Briefly, cells were plated into 96-well plates at a density of 3,000 cells/well. In each well, 10 μL of CCK-8 were added at various time points (0, 24, 48, and 72 h), and the cells were incubated at 37 °C for another 2 h. The absorbance at a wavelength of 450 nm was measured and used to generate cell growth curves.

### RIP assay

RIP assays were performed using a RIP RNA-binding protein immunoprecipitation kit (Millipore, USA) according to the manufacturer’s instruction. Briefly, the cells were lysed on ice-cold lysates supplemented with protease inhibitors, RNase inhibitors, and 1 mM PMSF and centrifuged at 1,500 rpm at 4 °C for 15 min. The protein extract (1 mg) was incubated with 3 μg of rabbit anti hnRNPA1 antibody (Cell Signaling Technology, USA) or rabbit IgG (Proteintech, USA) in reverse rotation at 4 °C overnight. Afterward, about 30 μL A/G protein beads were added, and the cells were suspended and incubated at 4 °C for 4 h. Thereafter, the beads were washed five times, and the co-immunoprecipitated miRNA was extracted by Ambion. The separated RNA was analyzed.

### Luciferase assay

MiRNA targets were predicted using TargetScan and miRDB. The cells (5 × 10^4^) were seeded into 24-well plates and cultured for 24 h. The reporter luciferase plasmid (100 ng), pGL3, pGL3–mut or control luciferase plasmid, and 5 ng pRL-TK *Renilla* plasmid (Promega, Madison, WI, USA) were transfected into the cells using Lipofectamine 3000 according to the manufacturer’s instructions. Luciferase and *Renilla* signals were measured 48 h after transfection using a Dual-Luciferase Reporter Assay Kit (Promega) according to the manufacturer’s protocol.

### Statistical analyses

Statistical analyses were performed using the Prism 5.0 (GraphPad Software, La Jolla, CA, USA) software. The results were presented as mean ± standard error of the mean (SEM). The Student’s *t*-test was used for the assessment of group differences. Differences with *P* < 0.05 were considered statistically significant.

## Results

### M2 macrophage infiltration is correlated with metastasis and a poor prognosis in HCC

**Fig. 1:**
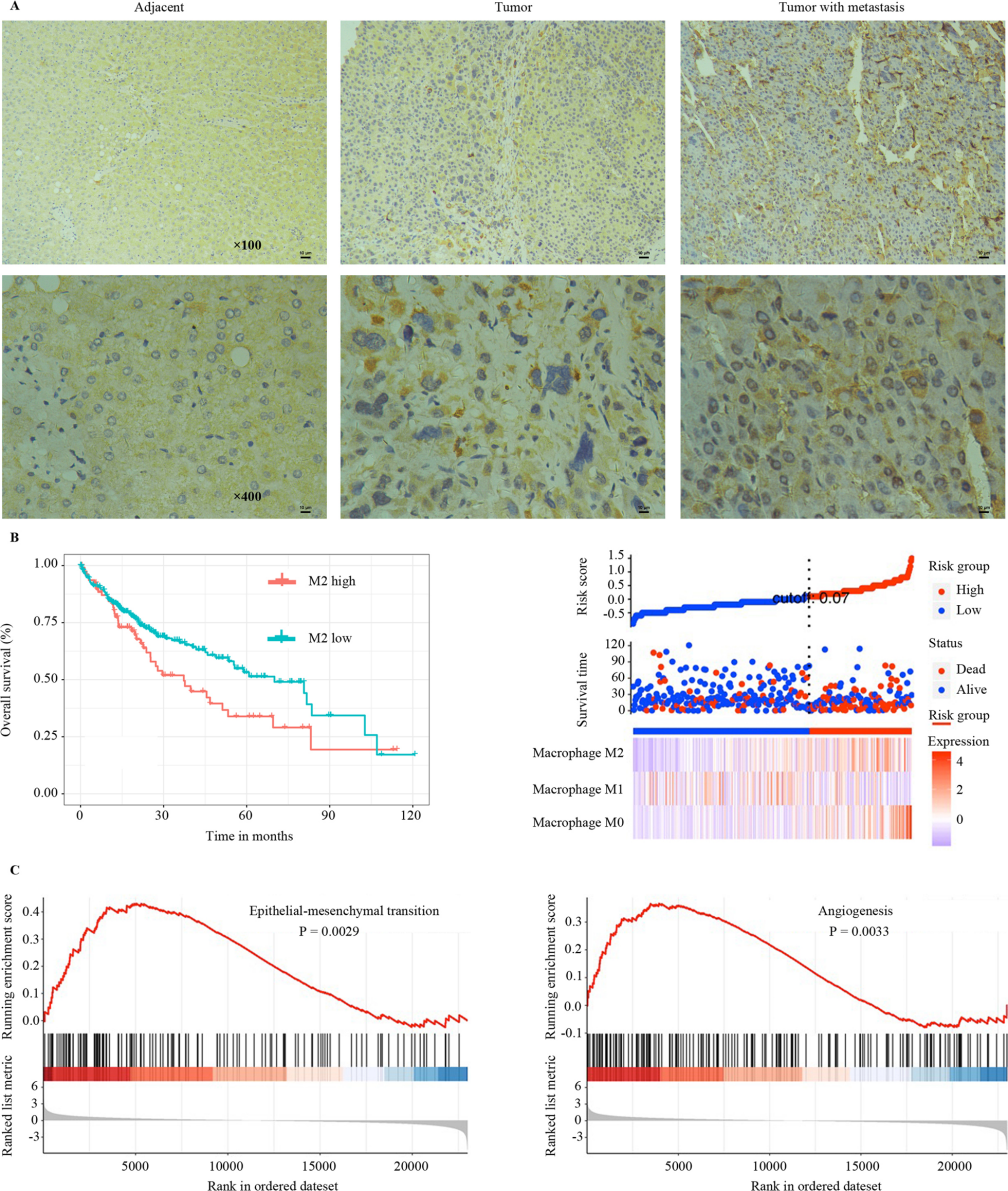
TAMs infiltration is correlated with metastasis and poor prognosis in HCC. **a** Immunohistochemistry of M2 macrophage marker CD163 in tumor tissues from HCC patients with or without metastasis. **b** Overall survival of CD163-positive macrophages in HCC patients. **c** GSEA was performed using TCGA HCC datasets to test the relationship between the expression of CCL2 in HCC tissues and EMT and angiogenesis

To study the relationship between M2 macrophage infiltration and HCC metastasis, the expression of CD163 (a marker of M2 macrophage) was assessed by immunohistochemistry in metastatic and non-metastatic HCC tissues. The results demonstrated that the CD163 expression level was significantly higher in metastatic HCC tissues (Fig. 1a). In addition, M2 macrophage infiltration was negatively correlated with the prognosis of HCC patients (Fig. 1b). To further explore the role of M2 macrophage-associated pathways in HCC metastasis, Gene Set Enrichment Analysis (GSEA) was carried out. The results revealed that EMT and angiogenesis were related to M2 macrophage infiltration (Fig. 1c). These results signal that M2 macrophage infiltration is correlated with HCC metastasis and prognosis and that M2 macrophages might have influenced HCC metastasis via EMT and angiogenesis.

### M2 macrophage-derived exosomes promote HCC cell metastasis and EMT

**Fig. 2:**
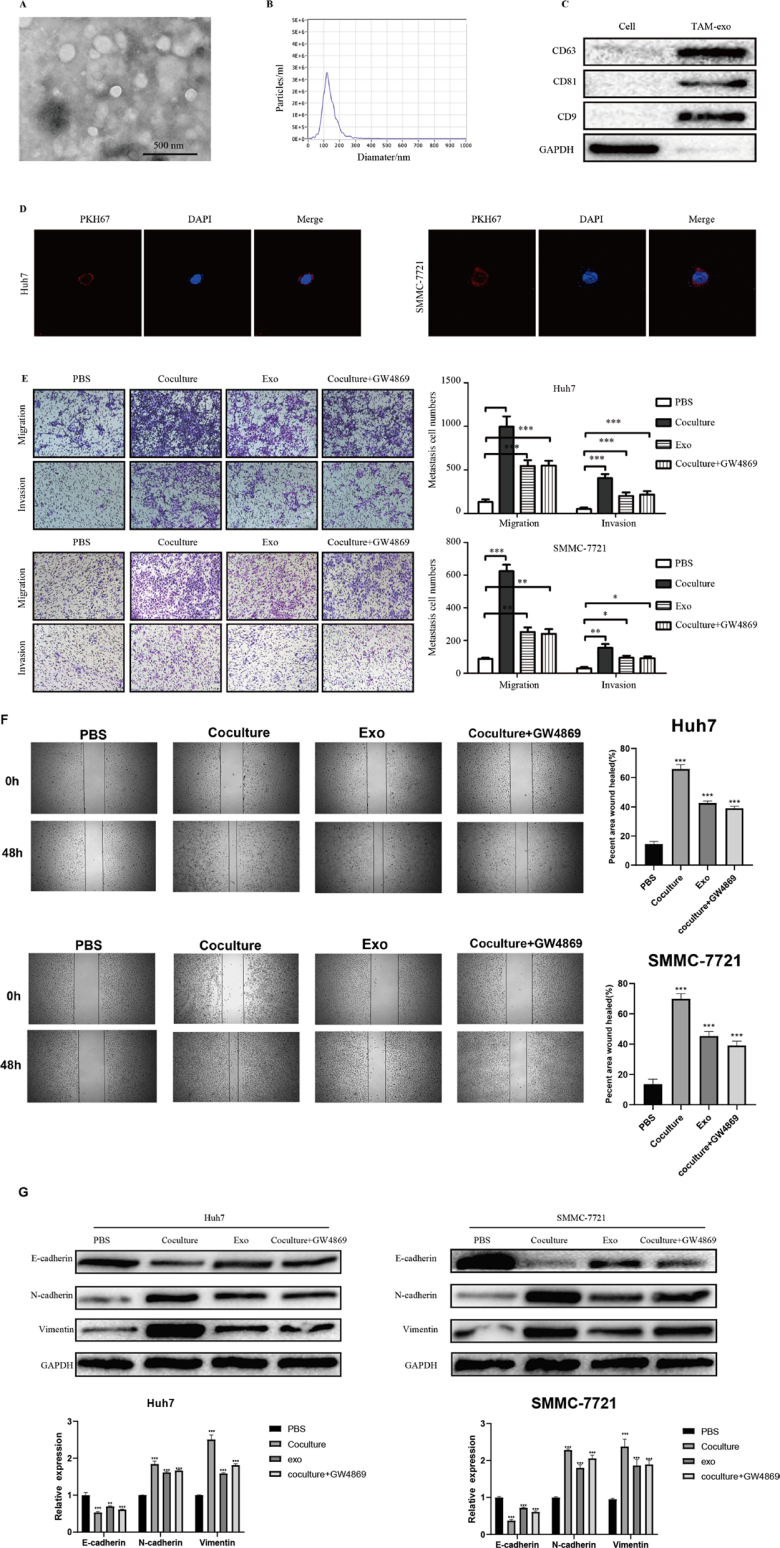
M2 macrophage exosomes promote HCC cell metastasis in vitro. **a** Transmission electron micrograph of exosomes in M2-polarized macrophages. **b** Exosome size distribution as analyzed by Nanosight particle tracking. **c** Western blot analysis of exosome marker CD63, CD81, and CD9 expression. **d** Exosome internalization by HCC cells was assessed using PKH67. **e** The effect of M2 macrophage derived exosomes on HCC cell metastasis as assessed using the Transwell® assay. **f**:The effect of M2 macrophage derived exosomes on HCC cell migratory ability as assessed using the the scratch assay. **g** The effect of M2 macrophage exosomes on HCC cell EMT was assessed by western blotting. *P < 0.05, **P < 0.01, ***P < 0.001

Human THP-1 monocytes were used to generate M2 macrophages. The polarized macrophages were confirmed by microscopy, flow cytometry, and immunofluorescence. The results exposed that M2 macrophages were spindle-shaped and expressed by CD163 (Supporting Additional file 1: Fig. S1a-c). Exosomes are cell signaling components that play an instrumental role in the tumor microenvironment. To further investigate whether exosomes participate in the regulation of HCC metastasis by macrophages, macrophage-derived exosomes were collected. The exosomes were cup-shaped, 50–100 nm in diameter, and expressed CD63, CD81, and CD9 (Fig. 2a-c). M2 macrophage-derived exosomes could be absorbed by HCC cells when co-cultured, as confirmed by PKH67 fluorescence staining (Fig. 2d). Figure 2e-f illustrates that co-culturing HCC cells with an M2 macrophage-conditioned medium enhanced migratory and invasive abilities. Besides, M2 exosomes alone also promoted HCC metastasis. When the M2 macrophage-conditioned medium was treated with GW4869, which inhibits the release of exosomes, the enhanced migratory and invasive abilities of HCC in the M2 macrophage-conditioned medium were reduced. These findings imply that M2 macrophage-derived exosomes promote HCC cell metastasis. As previously reported, M2 macrophages are correlated with EMT in HCC tissues. Consequently, the role of M2 macrophage-derived exosomes in the EMT of HCC cells was analyzed. Figure 2g depicts that M2 macrophage-derived exosomes reduce the expression of E-cadherin while increasing N-cadherin and vimentin expression. These findings indicate that M2 macrophage-derived exosomes promote metastasis and EMT of HCC cells.

### M2 macrophage-derived exosomes promote angiogenesis and increase vascular permeability

**Fig. 3:**
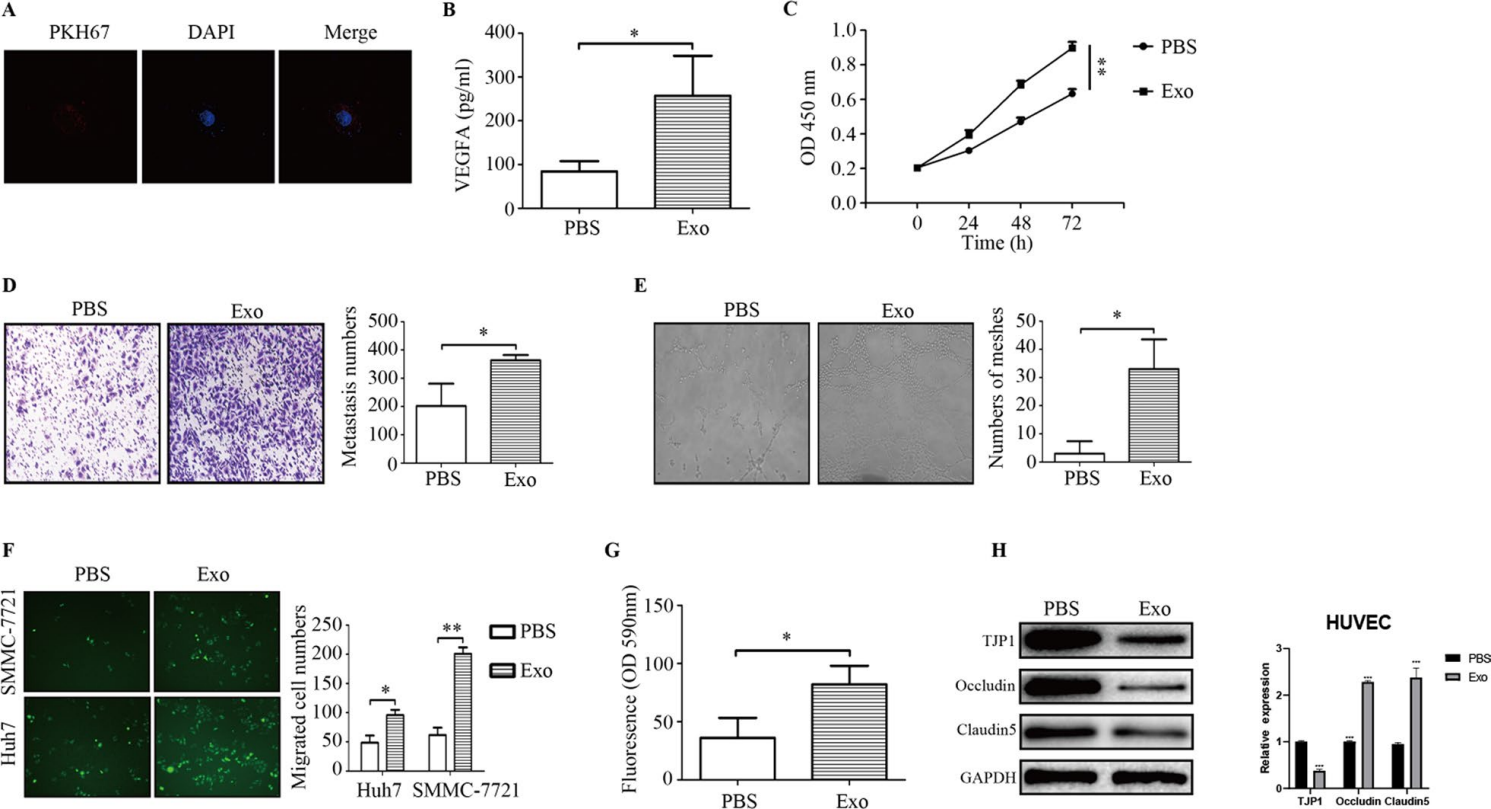
M2 macrophage exosomes promote angiogenesis and increase vascular permeability. **a** Exosomes internalization by HUVEC cells as assessed by PKH67. **b** The effect of M2 macrophage exosomes on the release of VEGFA as assessed by ELISA. **c d** The effect of M2 macrophage exosomes on the proliferation and migration of HUVECs as evaluated using the CCK-8 and Transwell^Ò^ assays. **e** The effect of M2 macrophage exosomes on angiogenesis as evaluated using a tube formation assay. **f, g** The effect of M2 macrophage exosomes on vascular permeability as tested by transendothelial invasion and endothelial permeability. **h** The effect of M2 macrophage exosomes on the expression of vascular adhesion molecules as assessed by western blotting. *P < 0.05, **P < 0.01,***P < 0.001

Angiogenesis and increased vascular permeability are vital components of tumor metastasis. M2 macrophages were correlated with angiogenesis in HCC tissues, as earlier described. Thus, the influence of M2 macrophage-derived exosomes on angiogenesis was further explored. Firstly, our results revealed that M2 macrophage-derived exosomes were internalized by HUVECs (Fig. 3a). Furthermore, M2 macrophage-derived exosomes increased the secretion of VEGFA and proliferation and metastasis of HUVECs (Fig. 3b–d). Afterward, a tube formation assay was conducted. Figure 3e delineates that M2 macrophage-derived exosomes enhanced angiogenesis. Intercellular adhesion is critical in maintaining endothelial integrity. Increased vascular permeability facilitates the penetration of tumor cells into blood vessels for distant metastasis. Herein, it was observed that M2 macrophage-derived exosomes downregulated the expression of TJP-1 and tight junction-related proteins occludin and claudin 5, and increased vascular permeability, indicating the number of GFP-positive cells that invaded through HUVEC monolayers and the amount of rhodamine B isothiocyanate-dextran (Fig. 3f–h). These findings highlight that M2 macrophage-derived exosomes promote angiogenesis and increase vascular permeability.

### M2 macrophage-secreted miR-23a-3p promotes HCC metastasis

**Fig. 4:**
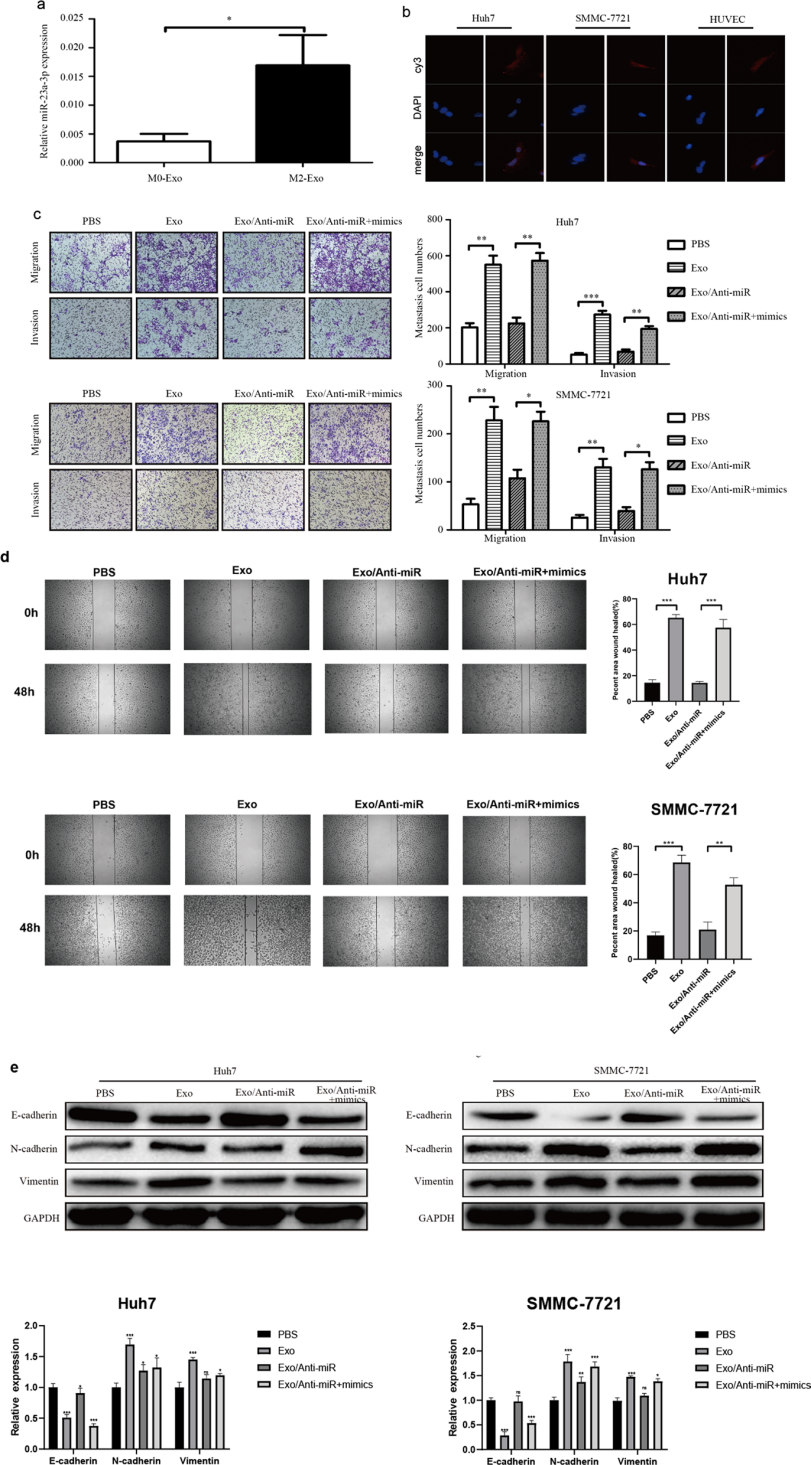
M2 macrophage exosomes transferred miR-23a-3p into HCC cells and HUVECs. **a** The expression of miR-23a-3p in M0 and M2 exosomes as tested by RT-PCR. **b** Cy3-miR-23a-3p internalization by HCC cells and HUVECs was evaluated by fluorescence microscopy. **c** The effect of M2 macrophage derived exosomal miR-23a-3p on HCC cell metastasis as assessed by the Transwell^Ò^ assay. **d**:The effect of M2 macrophage derived exosomal miR-23a-3p on HCC cell migratory ability as assessed by the scratch assay. **e** The effect of M2 macrophage exosomal miR-23a-3p on HCC cell EMT as evaluated by western blotting. *P < 0.05, **P < 0.01,***P < 0.001

Exosomes contain a large number of proteins and miRNAs, which are transferred to recipient cells. To determine the mechanism by which M2 macrophage-derived exosomes promote HCC metastasis, a miRNA microarray assay was performed. The results suggested that miR-23a-3p was upregulated in M2 macrophage-derived exosomes, which was validated by RT-PCR (Fig. 4a). Meanwhile, Cy3-miR-23a-3p internalization by HCC cells and HUVECs was also evaluated by fluorescence microscopy (Fig. 4b). When HCC cells and HUVECs were co-cultured with M2 macrophages, the expression of miR-23a-3p in HCC cells and HUVECs were upregulated. However, this upregulation was inhibited by GW4869, which inhibited the release of exosomes (Supporting Additional file 1: Fig. S2a). These findings signify that M2 macrophages transferred miR-23a-3p through exosomes. To investigate whether miR-23a-3p in exosomes plays a role in HCC metastasis, we decreased the expression of miR-23a-3p. When the expression of miR-23a-3p in M2 macrophages was knocked down (Supporting Additional file 1: Fig. S 2b), the expression of miR-23a-3p in M2 macrophage-derived exosomes also decreased (Supporting Additional file 1: Fig. S 2c). Furthermore, the upregulation of miR-23a-3p expression in HCC cells and HUVECs caused by M2 macrophage co-culture was also inhibited (Supporting Additional file 1: Fig. S2d). When miR-23a-3p was downregulated in exosomes, the effect of M2 exosomes on HCC metastasis, angiogenesis, and vascular permeability was lower, whereas an increase in miR-23a-3p expression restored the function of M2 macrophage exosomes (Fig. 4c-e and Supporting Additional file 1: Fig. S3).Meanwhile,the in vivo metasasiss assay also demonstrate the same trend(Supporting Additional file 1: Fig. S4).These findings indicate that M2 macrophage exosomes promote HCC metastasis by transferring miR-23a-3p.Meanwhile,the in vivo metastasis assay and the CAM assay also confirmed that the upregulation of miR-23a-3p expression in M2-exosomes promoted HCC metastasis and angiogenesis in vivo(Supporting Additional file 1: Fig. S4).

### hnRNPA1 mediates miR-23a-3p packaging into exosomes

**Fig. 5:**
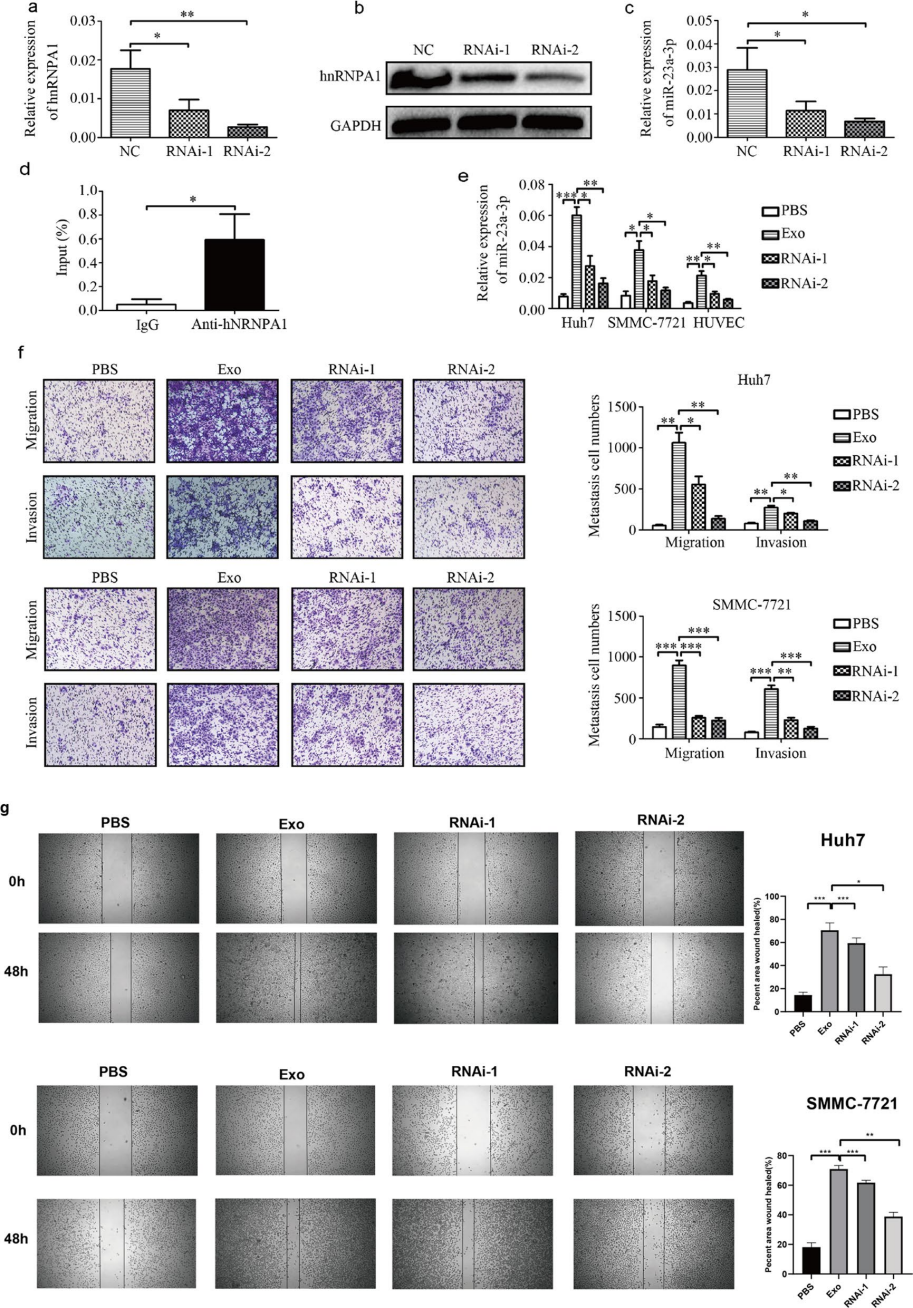
HnRNPA1 mediated miR-23a-3p packaging into M2 macrophage-derived exosomes. **a, b** Knockdown of hnRNPA1 was assessed by qRT-PCR and western blotting. **c** The effect of hnRNPA1 knock down of the expression of miR-23a-3p in M2 macrophage exosomes as analyzed by RT-PCR. **d** The combination of miR-23a-3p and hnRNPA1 was evaluated by the RIP assay. **e** The expression of miR-23a-3p in HCC cells and HUVECs when cocultured with M2 macrophages after hnRNPA1 knock down and assessed by RT-PCR. **f** The effect of M2 macrophage exosomes after hnRNPA1 knock down on HCC cell metastasis and evaluated by a Transwell^Ò^ assay. *P < 0.05, **P < 0.01, ***P < 0.001. **g**.The effect of M2 macrophage derived exosomes after hnRNPA1 knock down on HCC cell migratory ability as assessed by the scratch assay

To explore the process by which miR-23a-3p is packaged into exosomes, the binding proteins of miR-23a-3p were predicted using the database of RBP specificities (RBPDB, http://rbpdb.ccbr.utoronto.ca/; threshold 0.6). Among the top three binding proteins, we focused on hnRNPA1, considering that the hnRNP family has been reported to participate in microRNA packaging into exosomes. When hnRNPA1 was knocked down in macrophages (Fig. 5a-b), the expression of miR-23a-3p in exosomes was also reduced (Fig. 5c). Moreover, the connection between miR-23a-3p and hnRNPA1 was confirmed by RIP assays (Fig. 5d). When co-cultured with macrophages whose hnRNPA1 was knocked down, the upregulation of miR-23a-3p in HCC cells or HUVECs was also reversed (Fig. 5e). Subsequently, the effect of exosomes on HCC metastasis, angiogenesis, and vascular permeability after hnRNPA1 knockdown was investigated. The results showed that the promotion of M2 exosomes on HCC metastasis, angiogenesis, and vascular permeability was weaker with hnRNPA1 knockdown (Fig. 5f and Supporting Additional file 1: Fig. S5). These findings demonstrate that hnRNPA1-mediated miR-23a-3p sorting into exosomes promotes HCC metastasis, angiogenesis, and vascular permeability.

### HCC stimulated by M2 exosomes recruits more macrophages

**Fig. 6:**
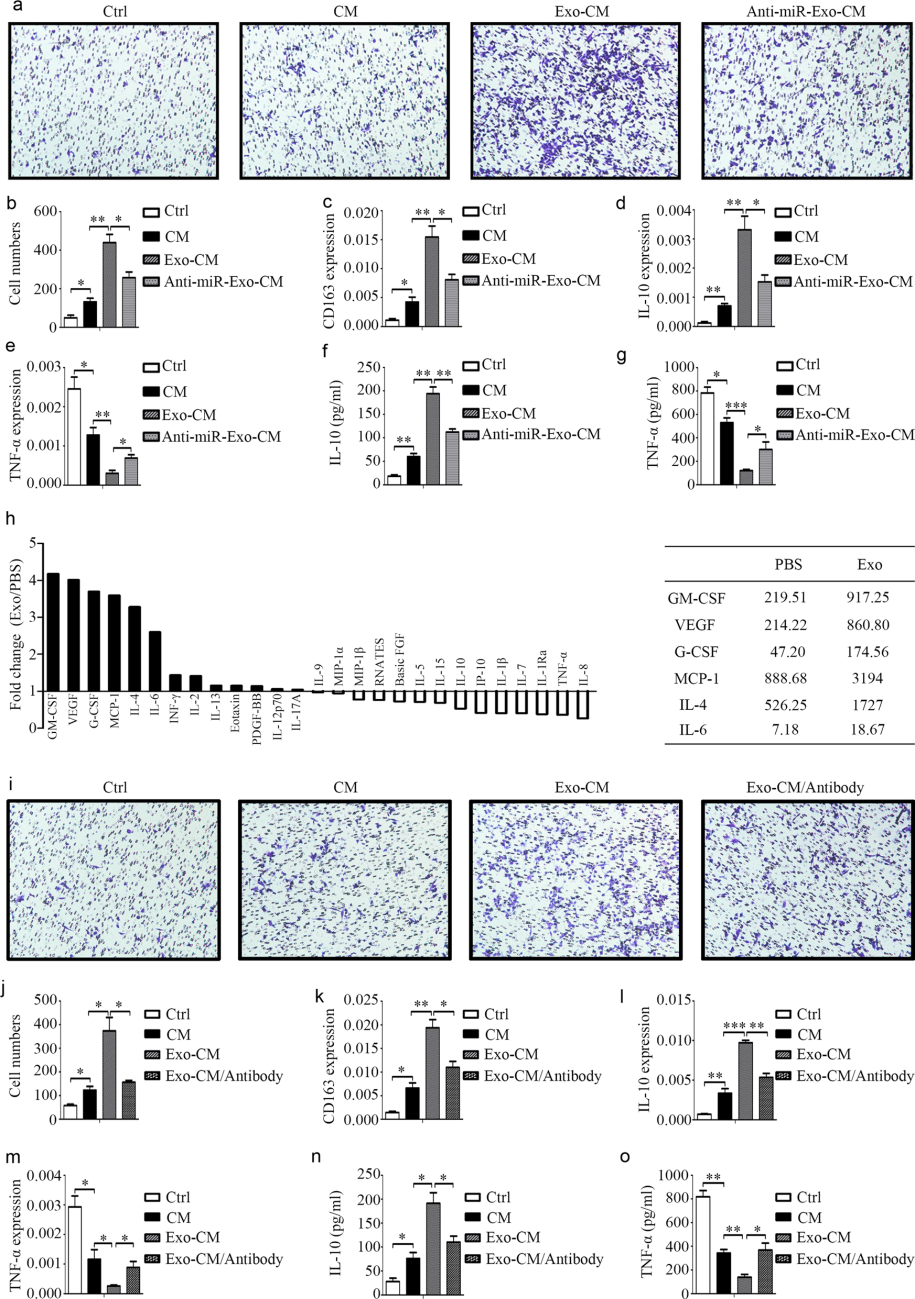
HCC cells induce macrophage recruitment and polarization. **a, b **Macrophage recruitment induced by HCC cells was assessed by a Transwell assay. **c** The expression of M2 macrophage marker CD163 in macrophages induced by HCC cells as tested by qRT-PCR. **d–g** The expression of IL-10 and TNF-αin macrophages induced by HCC cells as assessed by qRT-PCR and ELISA. **h** The effect of M2 macrophage exosomes on the secretion of CCL2 as evaluated by qRT-PCR and ELISA. **i**,**j** The effect of CCR2 knock down on macrophage recruitment as induced by HCC cells and evaluated by a Transwell^Ò^ assay. **k** The effect of CCR2 knock down on the expression of M2 macrophage marker CD163 in macrophages induced by HCC cells as assessed by qRT-PCR. **l–o** The effect of CCR2 knock down on the expression of IL-10 and TNF-αin macrophages induced by HCC cells and tested by qRT-PCR and ELISA. *P < 0.05, **P < 0.01, ***P < 0.001

In the tumor microenvironment, inflammatory cells can mediate the progression of tumor cells, and tumor cells can also recruit inflammatory cells and other cells. Thus, the effect of HCC cells on macrophages was explored. Figure 6a-g outline that HCC cells could recruit macrophages as well as induce M2 macrophage polarization. Furthermore, HCC cells treated with M2 macrophage-derived exosomes exhibited increased M2 macrophage recruitment and polarization, as reflected by the higher CD163 and IL-10 levels and lower TNF-α levels. In addition, the modulatory role of HCC cells on macrophage phenotypes was explored. Chemokines are a class of small cytokines or secreted signaling proteins that can induce directional chemotaxis of nearby responding cells. The mobilization and infiltration of inflammatory monocytes mainly depend on the CCL2/CCR2 signaling pathway. CCL2 is secreted by hepatocytes or hepatic stellate cells and is involved in liver injury, hepatitis, and liver cancer. Our results determined that HCC cells treated with M2 exosomes expressed higher levels of CCL2 (Fig. 6h). Then, the expression of CCR2, the only known receptor for CCL2, was knocked down. The results showed that when the expression of CCR2 was reduced in macrophages, the effect of HCC cells on M2 macrophage recruitment and polarization was diminished (Fig. 6i-o). These findings indicate that HCC cells treated with M2 macrophage-derived exosomes secrete increased amounts of CCL2 and further enhance M2 macrophage recruitment and polarization.

### PTEN and TJP1 are direct targets of miR-23a-3p

**Fig. 7:**
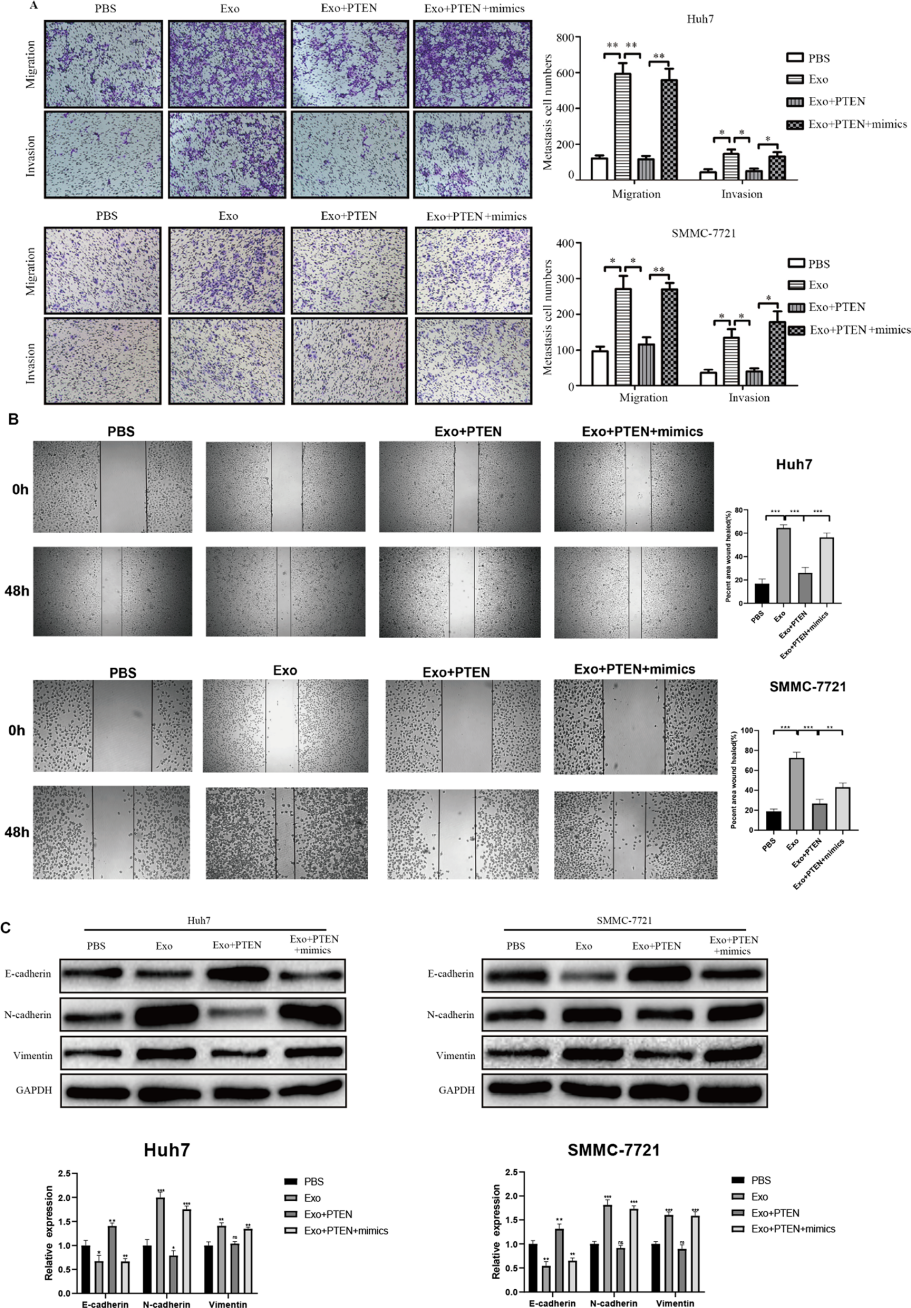
M2 macrophage exosomal miR-23a-3p promotes HCC cell metastasis and EMT by targeting PTEN. **a** The effect of PTEN on the metastasis of HCC cells induced by M2 macrophage exosomal miR-23a-3p and tested by a Transwell^Ò^ assay. **b**.The effect of PTEN on the on HCC cell migratory ability induced by M2 macrophage exosomal miR-23a-3p as assessed by the scratch assay.**c** The effect of PTEN on the EMT of HCC cells induced by M2 macrophage exosomal miR-23a-3p and tested by western blotting. *P < 0.05, **P < 0.01,***P < 0.001

TargetScan and miRDB were employed to identify the targets of miR-23a-3p. Among the potential targets, we focused on PTEN and TJP1. To verify their association, luciferase reporter experiments were performed. The results established that overexpression of miR-23a-3p reduced the luciferase activity of the wild-type 3'-UTR of PTEN and TJP1, but not the mutant (Supporting Additional file 1: Fig. S6a-d). Similarly, overexpression of miR-23a-3p reduced the expression of PTEN and TJP1 in HCC cells and HUVECs at the mRNA and protein levels (Supporting Additional file 1: Fig. S6e-h). Following this, the role of PTEN and TJP1 was investigated in HCC metastasis, angiogenesis, and vascular permeability. The HCC cells or HUVECs were transfected with PTEN and TJP1 before exosome incubation. The results demonstrated that PTEN and TJP1 overexpression reduced the effect of M2 exosomes on HCC metastasis, EMT, angiogenesis, and vascular permeability (Fig. 7 and Supporting Additional file 1: Fig. S7). However, restoration of miR-23a-3p eliminated the effect of PTEN and TJP1 overexpression. These findings suggest that M2 exosomes with miR-23a-3p promote HCC metastasis, angiogenesis, and vascular permeability by targeting PTEN or TJP1.

### High level of exosomal miR-23a-3p in the serum of HCC patients is related to poor prognosis

We selected 15 normal subjects and 40 HCC patients, and measured the level of miR-23a-3p in their serum exosomes. As shown in the figure, the level of miR-23a-3p in HCC patients was significantly higher than that in normal subjects(Supporting Additional file 1: Fig. S8a). Then we divided 40 HCC patients into two groups according to their expression levels, and plotted the overall survival time curve. The results showed that the high expression group predicted a poor prognosis(Supporting Additional file 1: Fig. S8b). Meanwhile, the clinical baseline table also suggested that high level of exosomal miR-23a-3p in the serum of HCC patients was correlated with age, tumor differentiation, tumor size, M stage, N stage and AJCC stage (Additional file 1: Table S1). These evidence suggest that the level of exosomal miR-23a-3p can be used as an index for the detection of HCC and was of important clinical implications.

## Discussion

TME plays a vital role in the occurrence and development of tumors. Exosomes are involved in signal transmission between cells in the tumor microenvironment. Studies have reported that exosomes derived from TAMs or other immune cells can promote tumor metastasis, drug resistance, and immune escape. This study determined that M2 macrophage-derived exosomes promote metastasis of HCC by transmitting miR-23a-3p.

Tumor metastasis is a complex, multi-step process. Firstly, tumor cells become more aggressive, and EMT is a key transition process for tumor cells to acquire high invasiveness. EMT is a biological process in which epithelial cells are transformed into phenotypic mesenchymal cells that are highly involved in chronic inflammation, tissue remodeling, tumor metastasis, and fibrotic diseases [18]. The cells are characterized by interstitial cells, including disrupted cell polarity and basement membrane junction, enhanced migration and invasion, upregulated anti-apoptosis, and extracellular matrix degradation [19, 20]. Studies have theorized that M2 exosomes promote tumor metastasis [21–23]. Whether M2 exosomes enhance EMT, particularly in HCC, warrants further investigation. Herein, M2 macrophage infiltration occurred with EMT in HCC. MiR-23a-3p was highly expressed in M2 macrophage-derived exosomes. It was transferred into HCC cells, which then exhibited reduced E-cadherin expression and increased N-cadherin and vimentin expression that promoted HCC cellular migration and invasion by targeting PTEN.

Tumor cells are separated from the primary tumor, invade the extracellular matrix, and are injected into blood vessels. Afterward, the “seeds” enter the target organs and develop to form metastatic nodules. Therefore, angiogenesis and increased vascular permeability play important roles in the second stage of tumor metastasis [24, 25]. The connection between vascular endothelial cells, such as VE-cadherin, ZO-1, and claudin-5, is essential to maintaining the integrity of vascular endothelial cells [26]. This study showed that loss of these adhesion molecules weakened endothelial connections and increased vascular permeability, thereby destroying the blood vessel barrier and triggering cancer cell metastasis. These findings indicate that tumor-derived exosomes enhance vascular permeability. HCC cells transferred miR-103 into HUVECs, target junction proteins, increased vascular permeability, and promoted HCC metastasis [17]. Hypoxia induces the generation of exosomes in lung cancer cells. In addition, exosomes under hypoxic conditions contain more miR-23a that directly targets PHD1 and PHD2, leading to increased angiogenesis and vascular permeability [27]. However, whether M2 exosomes influence angiogenesis and vascular permeability in HCC remains elusive. In this study, it was observed that M2 macrophage infiltration occurred with angiogenesis in HCC. Besides, M2 exosomes with miR-23a-3p increased the expression of VEGFA, the proliferation and metastasis of HUVEC cells, and angiogenesis by targeting PTEN. Moreover, M2 exosomes with miR-23a-3p downregulated TJP1, occludin, and claudin 5, leading to increased vascular permeability.

Recently, the pre-metastatic niche concept has become a research hotspot. The pre-metastatic niche, such as immunosuppression, abundant blood vessels, and inflammatory responses, provides a supportive microenvironment for disseminating tumor cells [28]. Thus, the pre-metastatic niche has become a route for studying tumor metastasis. Studies have exposed that exosomes participate in the formation of this pre-metastatic niche [29–31]. HCC cells with higher migratory and invasive abilities secrete miR-1247-3p-rich exosomes that activate cancer-associated fibroblasts (CAFs) to enhance HCC metastasis, invasion, and EMT via IL-6 and IL-8 [32]. However, the mechanism by which tumor cells stimulated by M2 exosomes recruit additional M2 macrophages to create a pre-metastatic niche for HCC metastasis remains unclear. In this study, tumor cells stimulated by M2 exosomes secreted more CCL2 and induced macrophage infiltration and M2 polarization. CCL2/CCR2 is a classic monocyte/macrophage recruitment signaling pathway that plays a pivotal role in HCC progression [33–35]. Blocking this pathway can inhibit inflammatory cell infiltration and M2 polarization, thereby reversing the immunosuppressive state of the tumor microenvironment and activating the anti-tumor CD8 + T cell response [36]. After knocking down the CCR2 receptor, the effect of the HCC cell supernatant on M2 macrophage infiltration was significantly reduced.

In short, our study postulates that hnRNPA1-mediated packaging of miR-23a-3p into M2 macrophage-derived exosomes promotes HCC cell metastasis by enhancing EMT, angiogenesis, and vascular permeability via targeting PTEN and TJP1. Moreover, HCC cells stimulated by M2-derived exosomes markedly enhanced M2 macrophage infiltration. Therefore, miR-23a-3p might serve as a potential therapeutic target for HCC metastasis.

## Conclusion

In summary, herein, a THP-1-induced M2 macrophage model was successfully constructed, which confirmed that it was associated with high infiltration, high metastatic rate, and a poor prognosis in HCC patients. Afterward, exosomes from M2 macrophages were extracted, and a series of functional experiments were conducted to verify its effect on HCC cell metastasis and angiogenesis. The subsequent miRNA microarray determined miR-23a-3p expression levels, which was significantly elevated in M2 macrophage-derived exosomes, and this high expression level was closely related to hnRNPA1-mediated miR-23a-3p packaging into exosomes. Luciferase reporter gene analysis validated that the targets of miR-23a-3p were phosphatase and tensin homolog (PTEN) and tight junction protein 1 (TJP1). Finally, the exosomes derived from M2 macrophages and hepatocellular carcinoma cells were co-cultured. The results showed that HCC cells secreted more GM-CSF, VEGF, G-CSF, MCP-1, and IL-4, and further recruited M2 macrophages.

### Supplementary Information

**Additional file 1.**

## Data Availability

All data in this study are available upon request.

## Abbreviations

HCC: Hepatocellular carcinoma
TAMs: Tumor-associated macrophages
HUVEC: Human umbilical vein endothelial cell
MiRNA: MicroRNA
EMT: Epithelial-mesenchymal transition
PTEN: Phosphatase and tensin homolog
TJP1: Tight junction protein 1
PMA: Phorbol-12-myristate-13 acetate
TEM: Transmission electron microscopy
PCR: Polymerase chain reaction
RIP: RNA immunoprecipitation
PMSF: Phenylmethylsulfonyl fluoride
GSEA: Gene set enrichment analysis
CAFs: Cancer-associated fibroblasts
