# Supplementary material for: M2 macrophage-secreted exosomes promote metastasis and increase vascular permeability in hepatocellular carcinoma
Source: Cell Commun Signal. 2023 Oct 30;21:299. doi: 10.1186/s12964-022-00872-w (PMC10614338; doi:10.1186/s12964-022-00872-w)
Supplement: Supplementary file 2 — Additional file 1. [file 12964_2022_872_MOESM1_ESM.pdf]

Table S1.The clinicopathological relevance analysis of the expression of exosomal miR-23a-3p in the serum of HCC patients.

Characteristic	Low group	High group	p
n	20	20	
gender, n (%)			0.333(ns)
Female	10 (25%)	6 (15%)	
Male	10 (25%)	14 (35%)	
Age, n (%)			0.041(*)
<55	10 (25%)	3 (7.5%)	
≥55	10 (25%)	17 (42.5%)	
Differentiation, n (%)			0.010(**)
poor	6 (15%)	15 (37.5%)	
Well to	14 (35%)	5 (12.5%)	
Tumor size, n (%)			0.010(**)
>5	7 (17.5%)	16 (40%)	
≤5	13 (32.5%)	4 (10%)	
N stage, n (%)			0.010(**)
N0	13 (32.5%)	4 (10%)	
N1	7 (17.5%)	16 (40%)	
M stage, n (%)			0.020(*)
M0	19 (47.5%)	12 (30%)	
M1	1 (2.5%)	8 (20%)	
AJCC stage, n (%)			<0.001(***)
stage I	10 (25%)	1 (2.5%)	
stage II	6 (15%)	0 (0%)	
stage III	3 (7.5%)	11 (27.5%)	

Characteristic	Low group	High group	p
stage IV	1 (2.5%)	8 (20%)	

Table S2. The primer sequences used in real-time PCR

Name	Sequence
IL-10	Forward:GTTGTTAAAGGAGTCCTTGCTG
	Reverse:TTCACAGGGAAGAAATCGATGA
TNF-α	Forward:CCTCTCTCTAATCAGCCCTCTG
	Reverse:GAGGACCTGGGAGTAGATGAG
CD163	Forward:TTTGTCAACTTGAGTCCCTTCAC
	Reverse:TCCCGCTACACTTGTTTTCAC
miR-23a-3p	ATCACATTGCCAGGGATTTCC
HnRNPA1	Forward:TCAGAGTCTCCTAAAGAGCCC
	Reverse:ACCTTGTGTGGCCTTGCAT
PTEN	Forward:TGGATTCGACTTAGACTTGACCT
	Reverse:GGTGGGTTATGGTCTTCAAAAGG
TJP1	Forward:CAACATACAGTGACGCTTCACA
	Reverse: CACTATTGACGTTTCCCCACTC
CCL2	Forward: CAGCCAGATGCAATCAATGCC
	Reverse: TGGAATCCTGAACCCACTTCT
CCR2	Forward: CCACATCTCGTTCTCGGTTTATC
	Reverse: CAGGGAGCACCGTAATCATAATC
GAPDH	Forward: GGAGCGAGATCCCTCCAAAAT
	Reverse: GGCTGTTGTCATACTTCTCATGG

Table S3. Antibody

Antibody	Assay	Product code	Dilution
CD163	IHC	Ab182422, Abcam, USA	1/500
CD63	WB	Ab134045, Abcam, USA	1/1000
CD81	WB	Ab109201, Abcam, USA	1/1000
CD9	WB	Ab195422, Abcam, USA	1:1000
E-cadherin	WB	#3195, CST, USA	1:1000
N-cadherin	WB	#13116, CST,USA	1:1000
Vimentin	WB	#5741, CST,USA	1:1000
TJP1	WB	#13663, CST, USA	1:1000
Occludin	WB	#91131, CST,USA	1:1000
Claudin5	WB	ab236066, Abcam, USA	1:1000
hnRNPA1	WB	#8443, CST, USA	1:1000
CCR2	WB	#12199, CST,USA	1:1000
GAPDH	WB	#5174, CST,USA	1:1000

Table S4. The sequences of siRNAs used in this study

Name	Sequence
miR-23a-3p mimics	5’-AUCACAUUGCCAGGGAUUUCC-3’
NC	5’-UUCUCCGAACGUGUCACGUTT-3’
miR-23a-3p Inhibitor	5’-UAGUGUAACGGUCCCUAAAGG-3’
NC	5’-GCUUAAGACAUUCCGAGGAAU-3’

Supporting Fig. legends

Supplementary Fig1. Polarized macrophage from Thp-1 was set up *in vitro*. **a:** The change of cell morphology was observed by microscope. **b, c:** Expression of CD68 and CD163 was determined by flow cytometry and IF.

Supplementary Fig2. Expression of miR-23a-3p in different groups was tested by RT-PCR. **a:** GW4869 decreased the upregulation of miR-23a-3p in HCC cells and HUVECs induced by M2 macrophage co-culture. **b:** Knockdown of miR-23a-3p in macrophages and tested by RT-PCR. **c:** The expression of miR-23a-3p in M2 macrophage exosomes after miR-23a-3p knockdown was induced by M2 macrophage exosomes or miR-23a-3p mimic and tested by RT-PCR. $^*P < 0.05$, $^{**}P < 0.01$, $^{***}P < 0.001$.**d**. The upregulation of miR-23a-3p expression in HCC cells and HUVECs caused by M2 macrophage coculture was also inhibited and tested by RT-PCR. $^*P < 0.05$, $^{**}P < 0.01$, $^{***}P < 0.001$.

Supplementary Fig3. M2 macrophage exosomes promote angiogenesis and increase vascular permeability through transferring miR-23a-3p. **a:** The effect of M2 macrophage exosomal miR-23a-3p on the release of VEGFA as determined by ELISA. **b, c:** The effect of M2 macrophage exosomal miR-23a-3p on the proliferation and migration of HUVECs was tested by the CCK-8 and Transwell ® assay. **d:** The effect of M2 macrophage exosomal miR-23a-3p on angiogenesis as tested by the tube formation assay. **e, f:** The effect of M2 macrophage exosomal miR-23a-3p on vascular permeability as tested by transendothelial invasion and endothelial permeability. **g:** The effect of M2 macrophage exosomal miR-23a-3p on the expression of vascular adhesion molecules as tested by western blotting. $^*P < 0.05$, $^{**}P < 0.01$,$^{***}P < 0.001$.

Supplementary Fig4. miR-23a-3p knockdown suppressed HCC cell metastasis and angiogenesis in vivo. A. Different groups of cells were injected into the caudal vein of nude mice to induce lung metastasis. typical bioluminescence images and lung tissues were obtained, and liver metastases were stained with HE.**B.**

Supplementary Fig5. HnRNPA1 mediates miR-23a-3p packaging into M2 macrophage-derived exosomes. **a:** The effect of hnRNPA1 knock down on the release of VEGFA induced by M2 exosomes and tested by ELISA. **b, c:** The effect of hnRNPA1 knock down on the proliferation and migration of HUVECs induced by M2 exosomes and tested by CCK-8 and Transwell® assay. **d:** The effect of hnRNPA1 knock down on angiogenesis induced by M2 exosomes and tested by a tube formation assay. **e, f:** The effect of hnRNPA1 knock down on vascular permeability induced by M2 exosomes and tested by transendothelial invasion and endothelial permeability. **g:** The effect of hnRNPA1 knock down on the expression of vascular adhesion molecules induced by M2 exosomes as tested by western blotting. *P < 0.05, **P < 0.01,***P < 0.001.

Supplementary Fig6. PTEN and TJP1 is the direct target of miR-23a-3p. **a** and **c:** The sequences of miR-23a-3p and the potential miR-23a-3p-binding sites at the 3'UTR of PTEN and TJP1 was predicted by targetscan. **b** and **d:** The effect of miR-23a-3p overexpression on the luciferase activity of PTEN and TJP1 was assessed using a luciferase assay. **e–h:** The effect of miR-23a-3p overexpression on the expression of PTEN and TJP1 was analyzed by qRT-PCR and western blotting. *P < 0.05, **P < 0.01, ***P < 0.001.

Supplementary Fig7. M2 macrophage exosomal miR-23a-3p promotes angiogenesis and increased vascular permeability by targeting PTEN and TJP1. **a:** The effect of PTEN on

VEGFA secretion cells induced by M2 macrophage exosomal miR-23a-3p and tested by ELISA. **b-c:** The effect of PTEN on the proliferation and migration of HUVECs induced by M2 macrophage exosomal miR-23a-3p and tested by CCK-8 and a Transwell® assay. **d:** The effect of PTEN on angiogenesis as induced by M2 macrophage exosomal miR-23a-3p and tested by the tube formation assay. **e, f:** The effect of TJP1 on vascular permeability as induced by M2 macrophage exosomal miR-23a-3p and tested by transendothelial invasion and endothelial permeability. **g:** The effect of TJP1 on the expression of vascular adhesion molecules as induced by M2 macrophage exosomal miR-23a-3p and tested by western blotting. *P < 0.05, **P < 0.01, ***P < 0.001.

Supplementary Fig8. High level of exosomal miR-23a-3p in the serum or plasma of HCC patients indicated a poor prognosis. a. Relative expression of exosomal miR-23a-3p in the serum of HCC patients and normal subjects was detected**. b.**The OS of two groups was plotted.

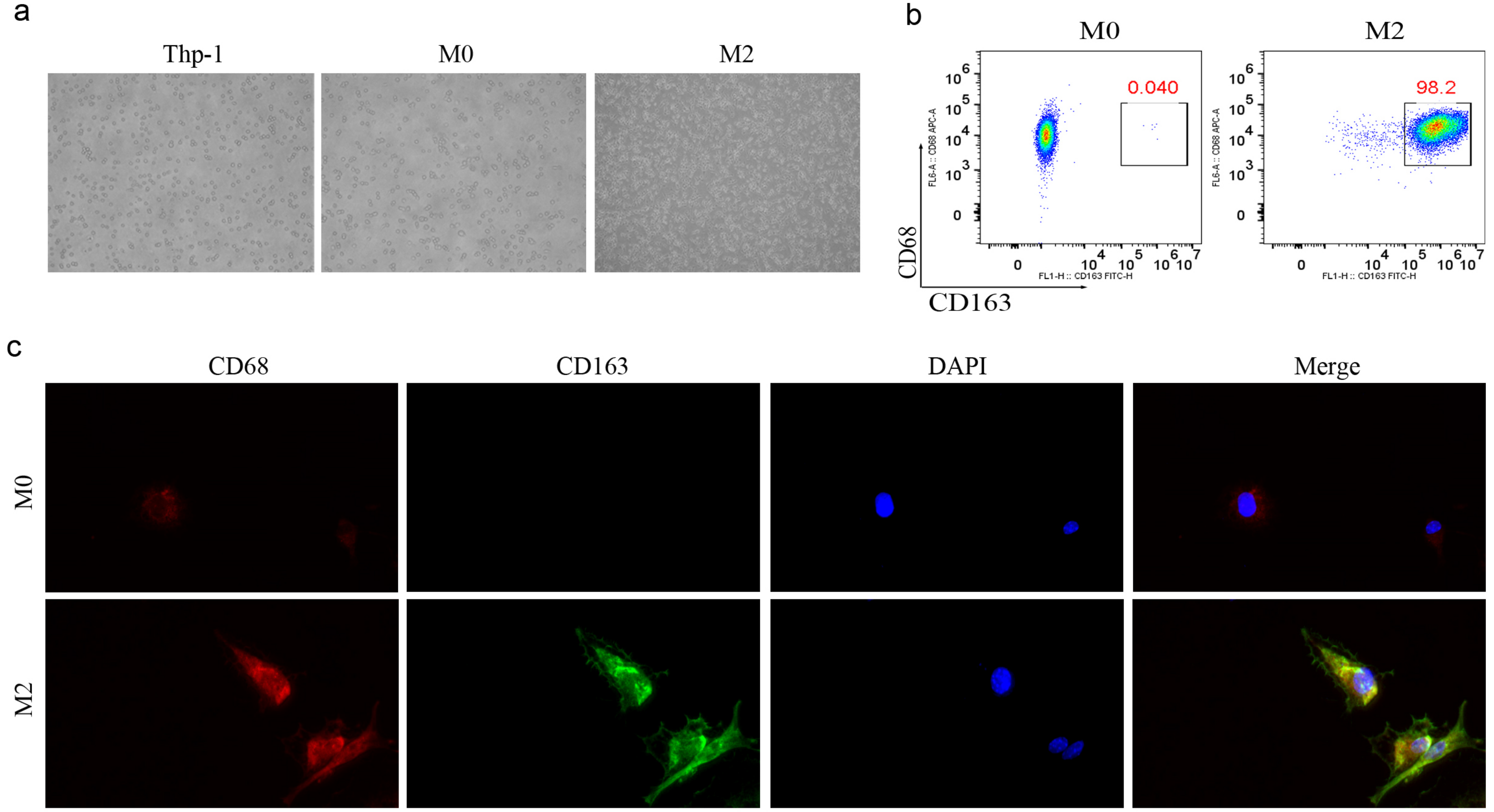
a
Thp-1
M0
M2
b
M0
M2
0.040
98.2
CD68
CD163
FL6-A :: CD68 APC-A
FL1-H :: CD163 FITC-H
c
CD68
CD163
DAPI
Merge
M0
M2

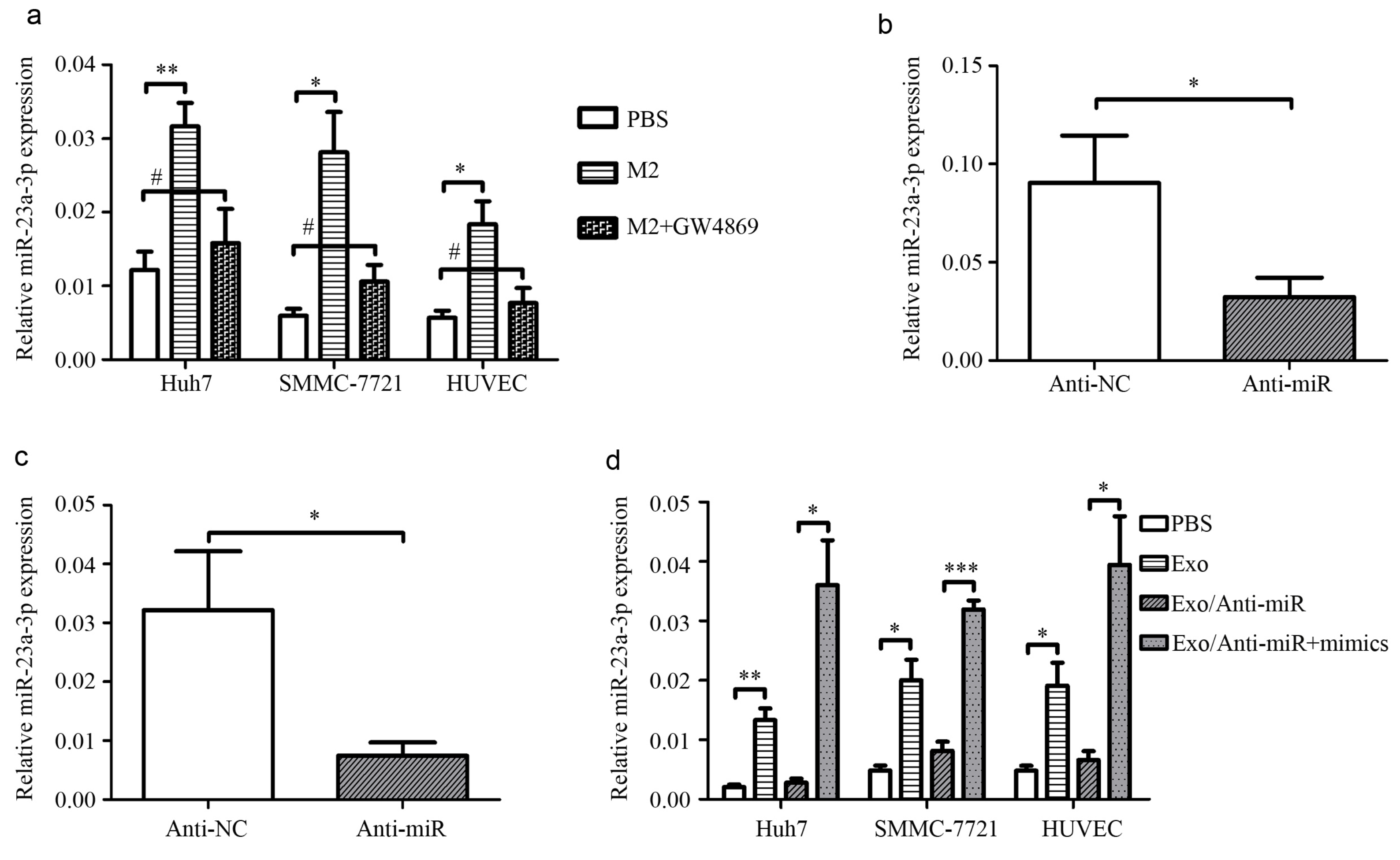

a
Relative miR-23a-3p expression
0.04
0.03
0.02
0.01
0.00
**
*
*
#
#
#
Huh7
SMMC-7721
HUVEC
PBS
M2
M2+GW4869
b
Relative miR-23a-3p expression
0.15
0.10
0.05
0.00
*
Anti-NC
Anti-miR
c
Relative miR-23a-3p expression
0.05
0.04
0.03
0.02
0.01
0.00
*
Anti-NC
Anti-miR
d
Relative miR-23a-3p expression
0.05
0.04
0.03
0.02
0.01
0.00
**
*
*
***
*
*
Huh7
SMMC-7721
HUVEC
PBS
Exo
Exo/Anti-miR
Exo/Anti-miR+mimics

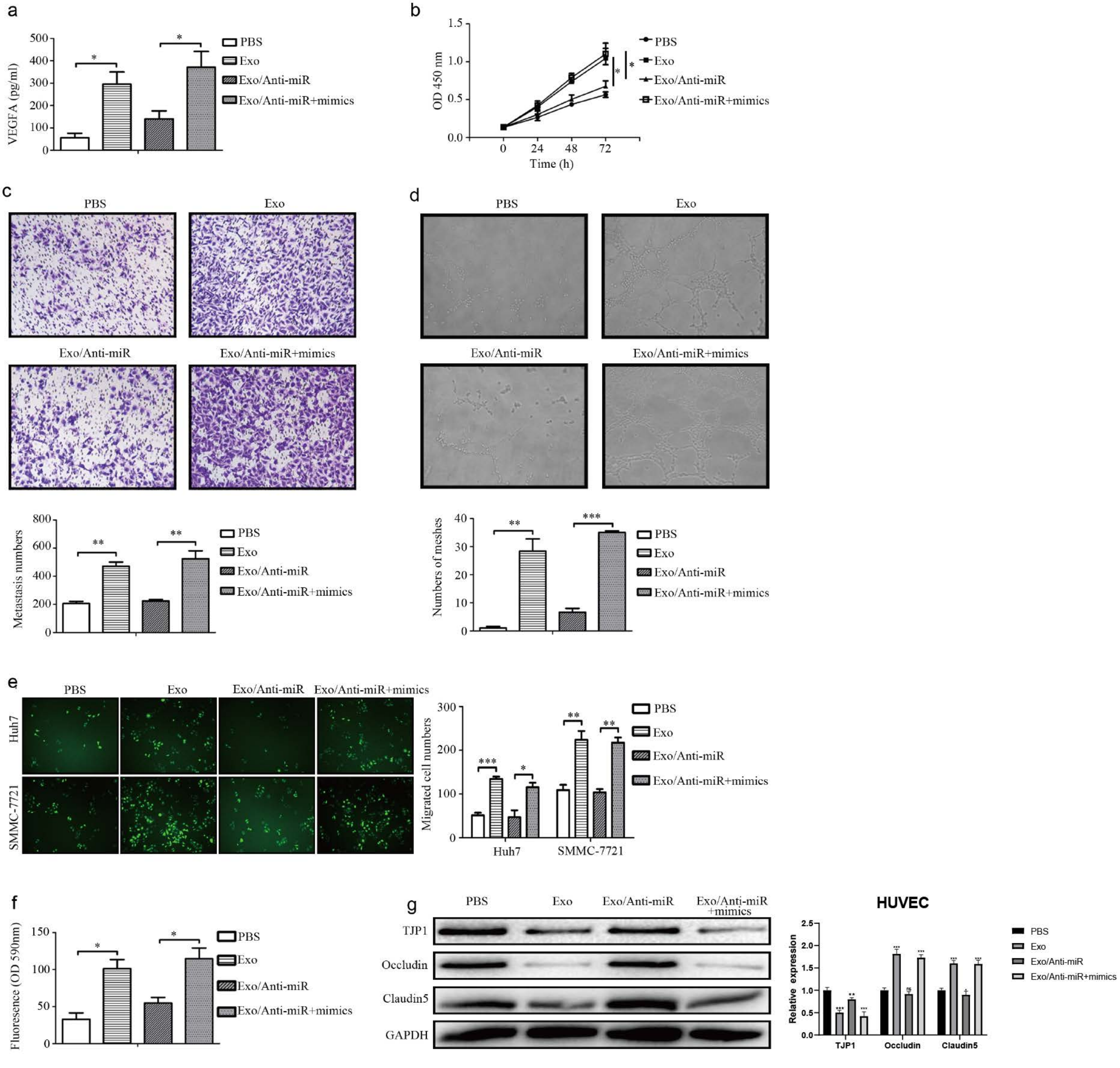
a
VEGFA (pg/ml)
PBS
Exo
Exo/Anti-miR
Exo/Anti-miR+mimics
b
OD 450 nm
Time (h)
PBS
Exo
Exo/Anti-miR
Exo/Anti-miR+mimics
c
PBS
Exo
Exo/Anti-miR
Exo/Anti-miR+mimics
Metastasis numbers
d
PBS
Exo
Exo/Anti-miR
Exo/Anti-miR+mimics
Numbers of meshes
e
PBS
Exo
Exo/Anti-miR
Exo/Anti-miR+mimics
Huh7
SMMC-7721
Migrated cell numbers
f
Fluoresence (OD 590nm)
PBS
Exo
Exo/Anti-miR
Exo/Anti-miR+mimics
g
PBS
Exo
Exo/Anti-miR
Exo/Anti-miR +mimics
TJP1
Occludin
Claudin5
GAPDH
HUVEC
Relative expression
TJP1
Occludin
Claudin5
PBS
Exo
Exo/Anti-miR
Exo/Anti-miR+mimics

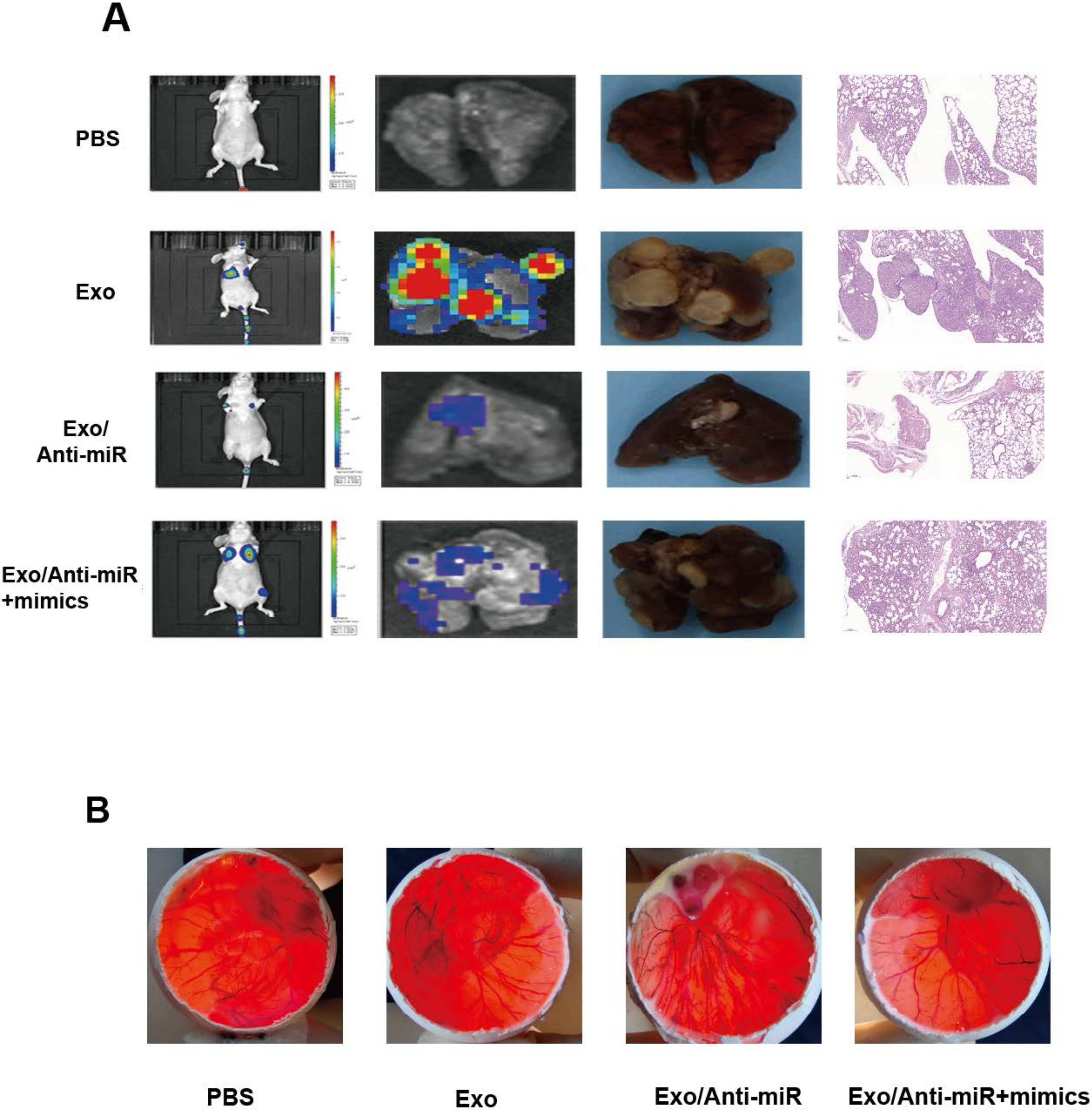
A
PBS
Exo
Exo/
Anti-miR
Exo/Anti-miR
+mimics
B
PBS
Exo
Exo/Anti-miR
Exo/Anti-miR+mimics

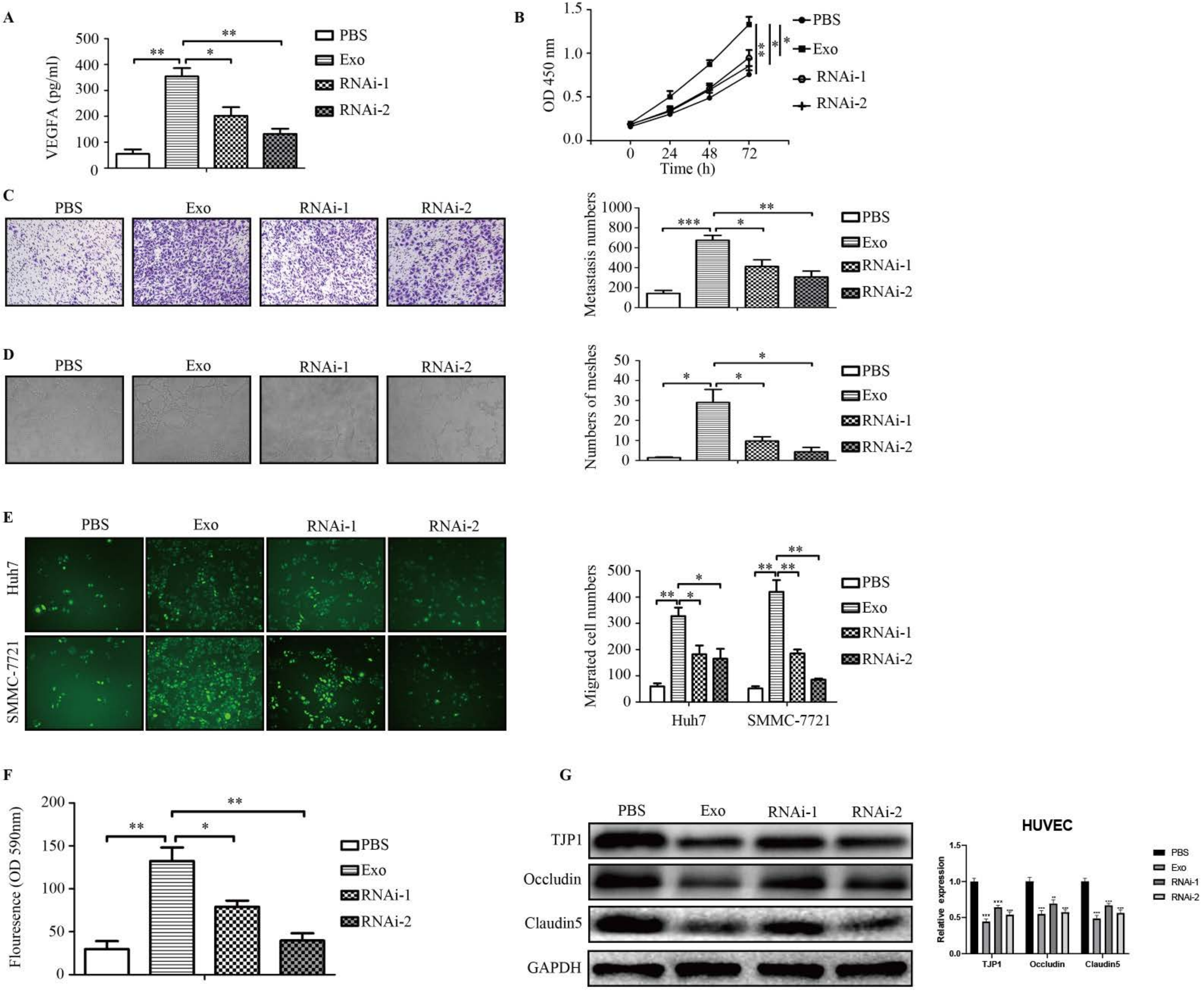
A
VEGFA (pg/ml)
500
400
300
200
100
0
PBS
Exo
RNAi-1
RNAi-2
B
OD 450 nm
1.5
1.0
0.5
0.0
0
24
48
72
Time (h)
PBS
Exo
RNAi-1
RNAi-2
C
PBS
Exo
RNAi-1
RNAi-2
Metastasis numbers
1000
800
600
400
200
0
PBS
Exo
RNAi-1
RNAi-2
D
PBS
Exo
RNAi-1
RNAi-2
Numbers of meshes
50
40
30
20
10
0
PBS
Exo
RNAi-1
RNAi-2
E
PBS
Exo
RNAi-1
RNAi-2
Huh7
SMMC-7721
Migrated cell numbers
500
400
300
200
100
0
Huh7
SMMC-7721
PBS
Exo
RNAi-1
RNAi-2
F
Flouresence (OD 590nm)
200
150
100
50
0
PBS
Exo
RNAi-1
RNAi-2
G
PBS
Exo
RNAi-1
RNAi-2
TJP1
Occludin
Claudin5
GAPDH
HUVEC
Relative expression
1.5
1.0
0.5
0.0
TJP1
Occludin
Claudin5
PBS
Exo
RNAi-1
RNAi-2

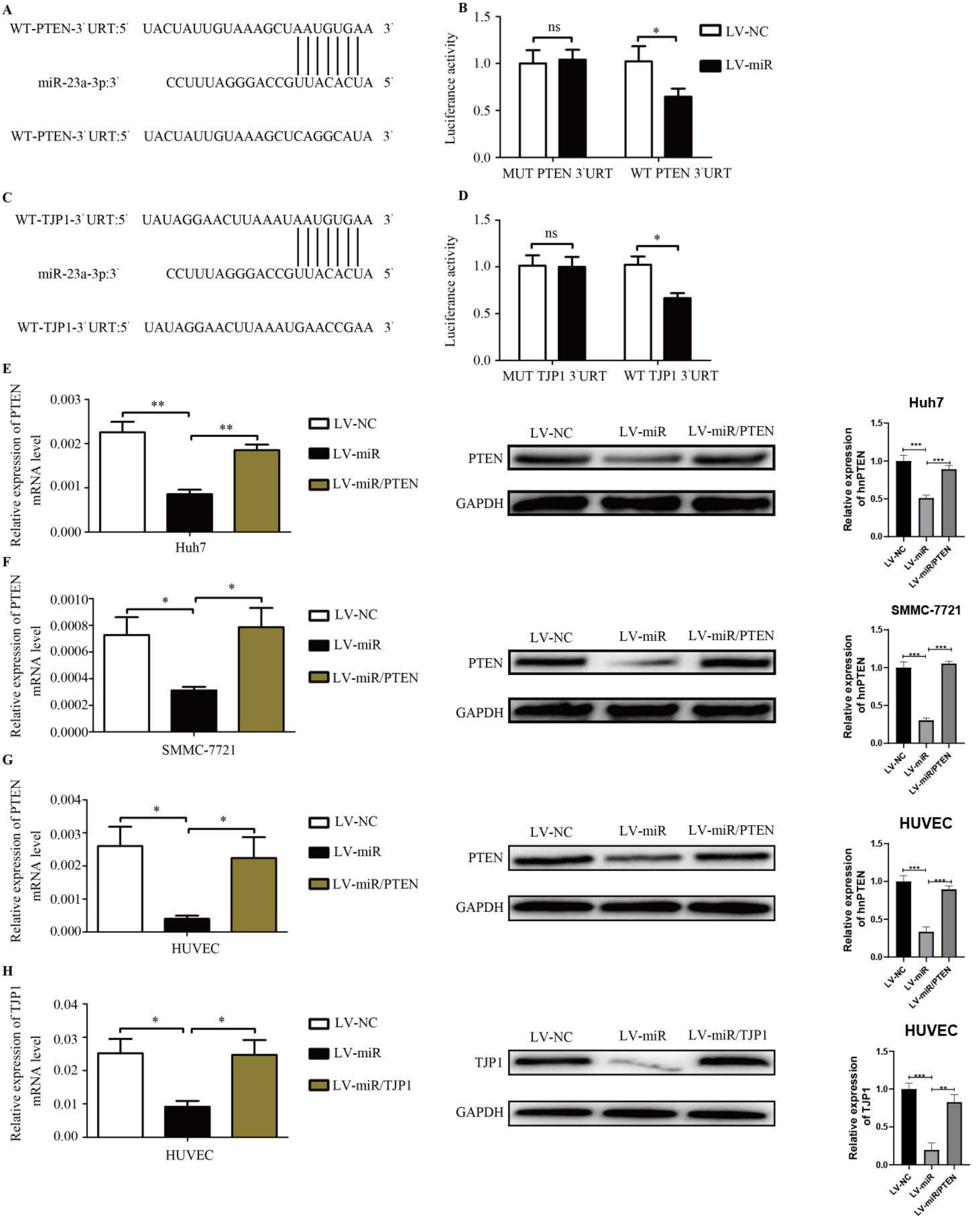
A
WT-PTEN-3`URT:5` UACUAUUGUAAAGCUAAUGUGAA 3`
miR-23a-3p:3` CCUUUAGGGACCGUUACACUA 5`
WT-PTEN-3`URT:5` UACUAUUGUAAAGCUCAGGCAUA 3`
B
Luciferance activity
LV-NC
LV-miR
ns
*
MUT PTEN 3`URT
WT PTEN 3`URT
C
WT-TJP1-3`URT:5` UAUAGGAACUUAAAUAAUGUGAA 3`
miR-23a-3p:3` CCUUUAGGGACCGUUACACUA 5`
WT-TJP1-3`URT:5` UAUAGGAACUUAAAUGAACCGAA 3`
D
Luciferance activity
MUT TJP1 3`URT
WT TJP1 3`URT
E
Relative expression of PTEN mRNA level
Huh7
LV-NC
LV-miR
LV-miR/PTEN
PTEN
GAPDH
Relative expression of hnPTEN
F
SMMC-7721
G
HUVEC
H
Relative expression of TJP1 mRNA level
LV-miR/TJP1
TJP1
Relative expression of TJP1

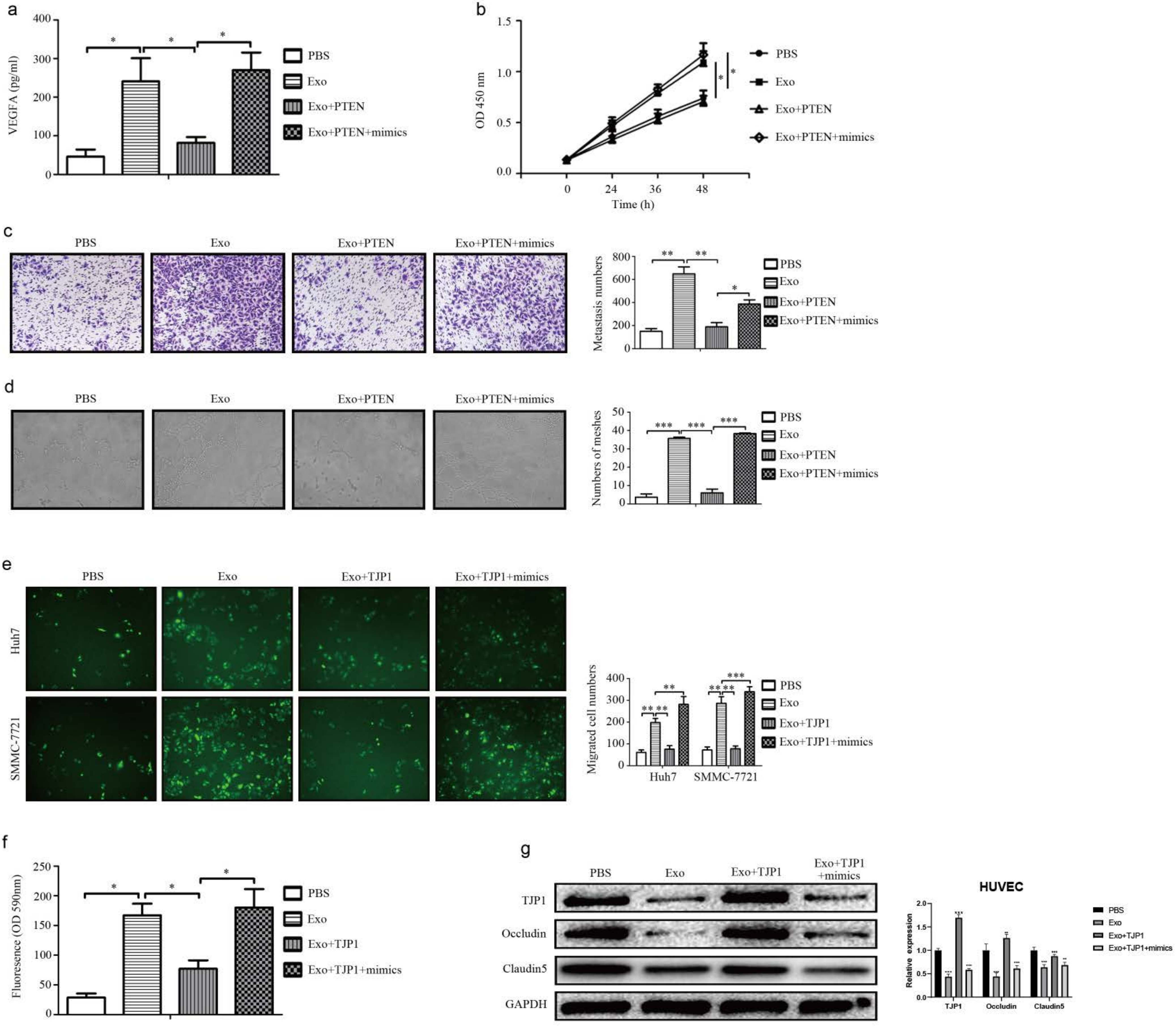

a
VEGFA (pg/ml)
PBS
Exo
Exo+PTEN
Exo+PTEN+mimics
b
OD 450 nm
Time (h)
PBS
Exo
Exo+PTEN
Exo+PTEN+mimics
c
PBS
Exo
Exo+PTEN
Exo+PTEN+mimics
Metastasis numbers
d
PBS
Exo
Exo+PTEN
Exo+PTEN+mimics
Numbers of meshes
e
PBS
Exo
Exo+TJP1
Exo+TJP1+mimics
Huh7
SMMC-7721
Migrated cell numbers
f
Fluoresence (OD 590nm)
PBS
Exo
Exo+TJP1
Exo+TJP1+mimics
g
PBS
Exo
Exo+TJP1
Exo+TJP1 +mimics
TJP1
Occludin
Claudin5
GAPDH
HUVEC
Relative expression
PBS
Exo
Exo+TJP1
Exo+TJP1+mimics

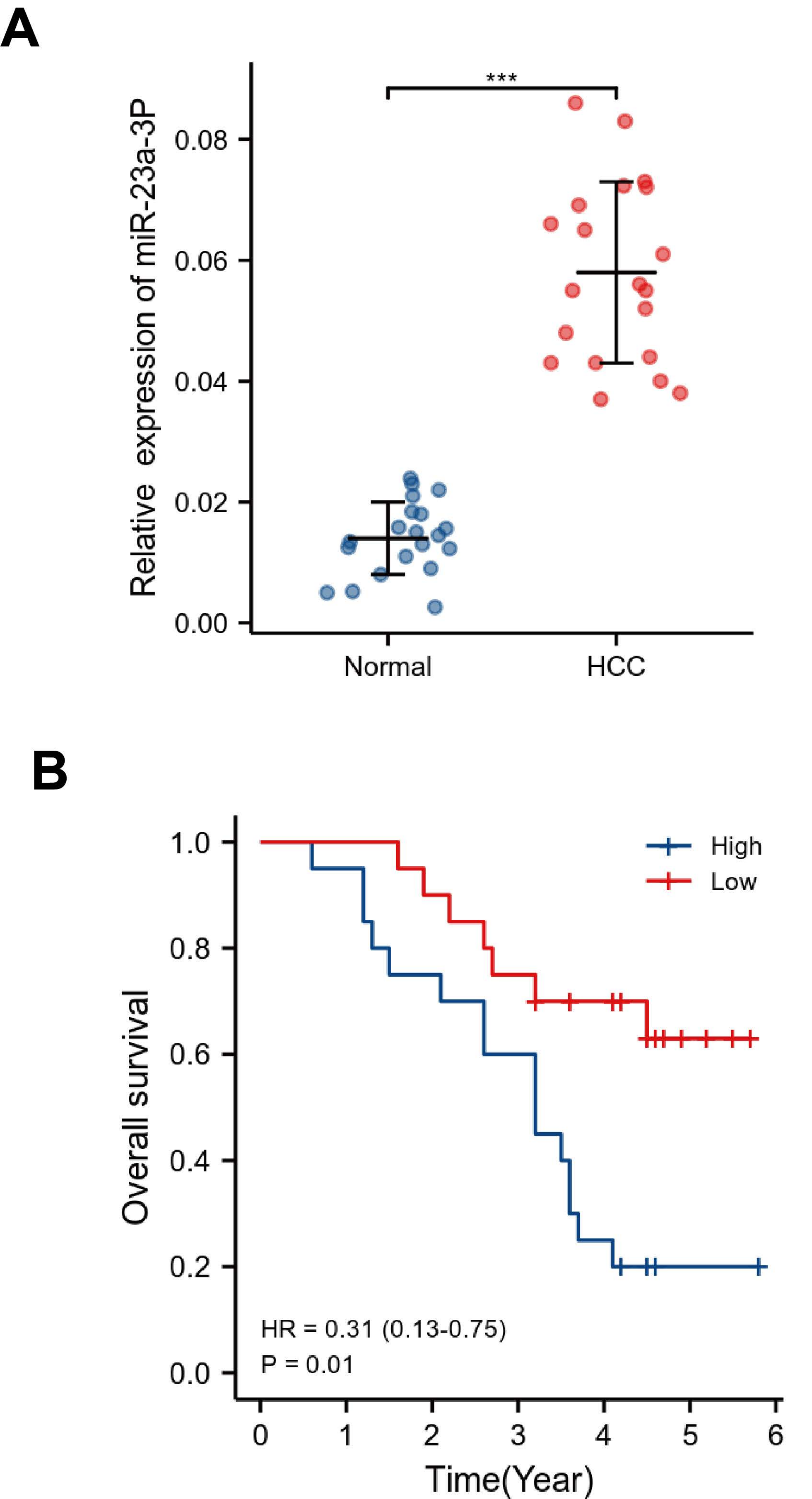
A
***
Relative expression of miR-23a-3P
0.08
0.06
0.04
0.02
0.00
Normal
HCC
B
High
Low
1.0
0.8
0.6
0.4
0.2
0.0
Overall survival
HR = 0.31 (0.13-0.75)
P = 0.01
0
1
2
3
4
5
6
Time(Year)
